# Supplementary material for: Projecting vaccine demand and impact for emerging zoonotic pathogens
Source: BMC Med. 2022 Jun 16;20:202. doi: 10.1186/s12916-022-02405-1 (PMC9200440; doi:10.1186/s12916-022-02405-1)
Supplement: Supplementary file 1 — Additional file 1: Table S1. Overview of data references. Table S2. Sizes of single reactive vaccination campaigns targeting the general population or healthcare workers (HCWs). SI Text. Sensitivity analysis and extended model limitations. Fig. S1. Spillover and reactive vaccination patterns for Lassa fever virus (LASV). Fig. S2. Spillover and reactive vaccination patterns for Middle Eastern respiratory virus (MERS-CoV). Fig. S3. Spillover and reactive vaccination patterns for Nipah virus (NiV). Fig. S4. Spillover and reactive vaccination patterns for Rift Valley fever virus (RVFV). Fig. S5. Vaccine regimens required for Lassa fever virus (LASV). Fig. S6. Vaccine regimens required for Middle Eastern respiratory virus (MERS-CoV). Fig. S7. Vaccine regimens required for Nipah virus (NiV). Fig. S8. Vaccine regimens required for Rift Valley fever virus (RVFV). Fig. S9. Vaccine regimens required to vaccinate healthcare workers for Lassa fever virus (LASV). Fig. S10. Vaccine regimens required to vaccinate healthcare workers for Middle Eastern respiratory virus (MERS-CoV). Fig. S11. Vaccine regimens required to vaccinate healthcare workers for Nipah virus (NiV). Fig. S12. Vaccine regimens required to vaccinate veterinarians for Rift Valley fever virus (RVFV). Fig. S13. Vaccination impact sensitivity analysis for LASV. Fig. S14. Vaccination impact sensitivity analysis for NiV. Fig. S15. Vaccination impact sensitivity analysis for RVFV. Fig. S16. Number of cases under different R0 assumptions. Fig. S17. Number of vaccine regimens required under different R0 assumptions. Fig. S18. Number of vaccine regimens required for healthcare workers (HCWs) under different R0 assumptions. Fig. S19. Number of cases averted by vaccinating the general population under different R0 assumptions. Fig. S20. Fraction of cases averted by vaccinating the general population under different R0 assumptions. Fig. S21. Number of cases averted per vaccine regimen administered to the general population un [file 12916_2022_2405_MOESM1_ESM.docx]

**Supplementary Information**

**Table of Contents**

Table S1 ………………………...…….…………………………………………………………...…..... 2

Table S2 ……………………………………………………………………………………………...….. 3

Figures S1-S4 …………………...…………………………………………………………….….…….. 4

SI Text ……………………………………………………….…………………………………………… 8

1. Sensitivity analysis: Reactive vaccination strategies …..…………..………..…………….. 8
2. Sensitivity analysis: R_0_ ………………………………………….…...…...………..………….. 9
3. Model limitations …………………………………………………..….………………………. 10
   1. Figures S5-S22 …………..…………………………………………………………... 16
4. Analysis of different spillover and vaccination catchment areas ………………………… 37

Figures S23-S34 ……………………………………………………………….………………………. 38

References ……………………………………………………………………………………………... 54

**Table S1. Overview of data references.**

| **Parameter** | **LASV** | **MERS-CoV** | **NiV** | **RVFV** |
| --- | --- | --- | --- | --- |
| Case reports | 1-11 | 11-14 | 11,15-21 | 11,22-41 |
| Seasonality | See case reports | See case reports | See case reports | See case reports |
| Incubation period | 42-45 | 46-49 | 18 | 50-57 |
| Infectious period | 45 | 46,49,58,59 | 60 | 61 |
| R_0_ | 62 | 47, 63-67 | 18 |  |

**Table S2. Sizes of single reactive vaccination campaigns targeting the general population or healthcare workers (HCWs).** Median campaign sizes with 95% prediction interval (PrI) in parentheses.

| **Pathogen** | **General Population** | **Healthcare workers (HCWs)** |
| --- | --- | --- |
| LASV | 153,773  (47,732 – 485,034) | 282  (88 – 890) |
| MERS-CoV | 275,471  (90,171 – 358,259) | 2,149  (704 – 2608) |
| NiV | 460,408  (32,633 – 5,098,459) | 545  (182 – 9,971) |
| RVFV | 156,634  (1,478 – 1,162,080) | 159  (0 – 5,699) |

**
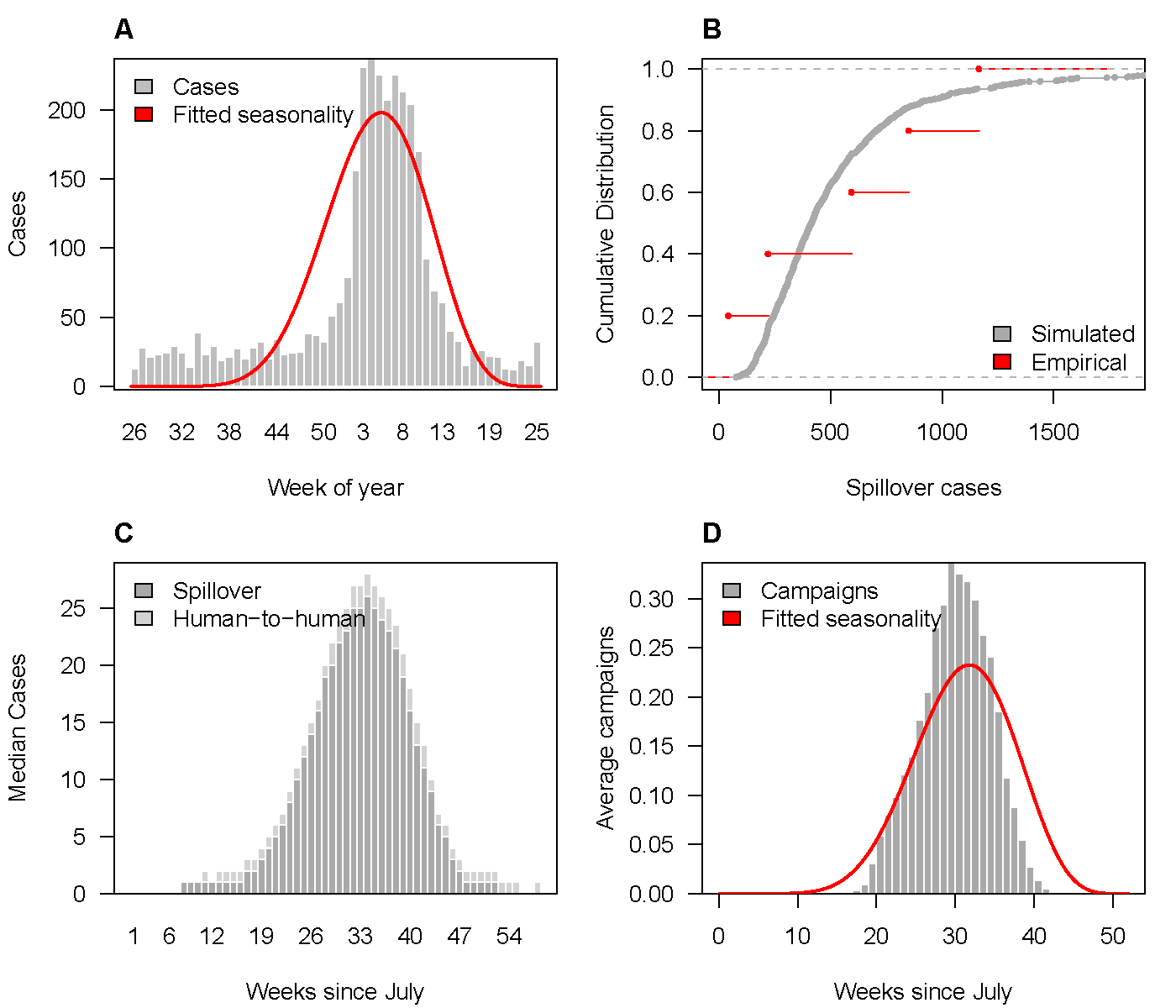
**

**Figure S1. Spillover and reactive vaccination patterns for Lassa fever virus (LASV).** (A) Observed weekly Lassa fever spillover cases (grey bars) and estimated seasonal spillover rate (red line). (B) Annual number of spillovers over the past 5 years (red) and cumulative distribution of simulated annual spillovers from 1000 replicates (grey). (C) Median weekly simulated spillover and human-to-human Lassa fever cases. (D) Average weekly number of reactive campaigns triggered via spillover detection compared to the estimated seasonal spillover rate (red line).

**
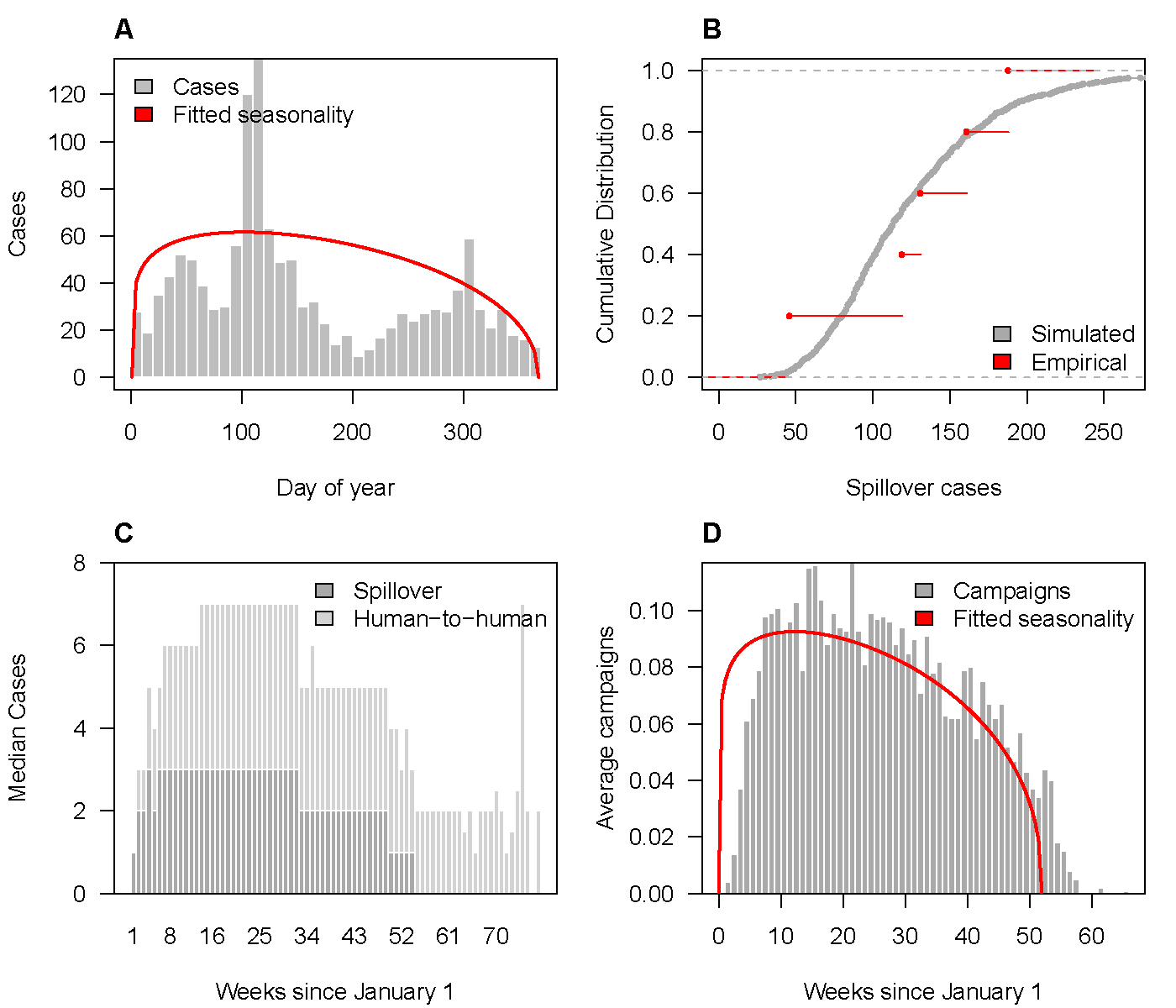
**

**Figure S2. Spillover and reactive vaccination patterns for Middle Eastern respiratory virus (MERS-CoV).** (A) Observed weekly MERS spillover cases (grey bars) and estimated seasonal spillover rate (red line). (B) Annual number of spillovers over the past 5 years (red) and cumulative distribution of simulated annual spillovers from 1000 replicates (grey). (C) Median weekly simulated spillover and human-to-human MERS cases. (D) Average weekly number of reactive campaigns triggered via spillover detection compared to the estimated seasonal spillover rate (red line).

**
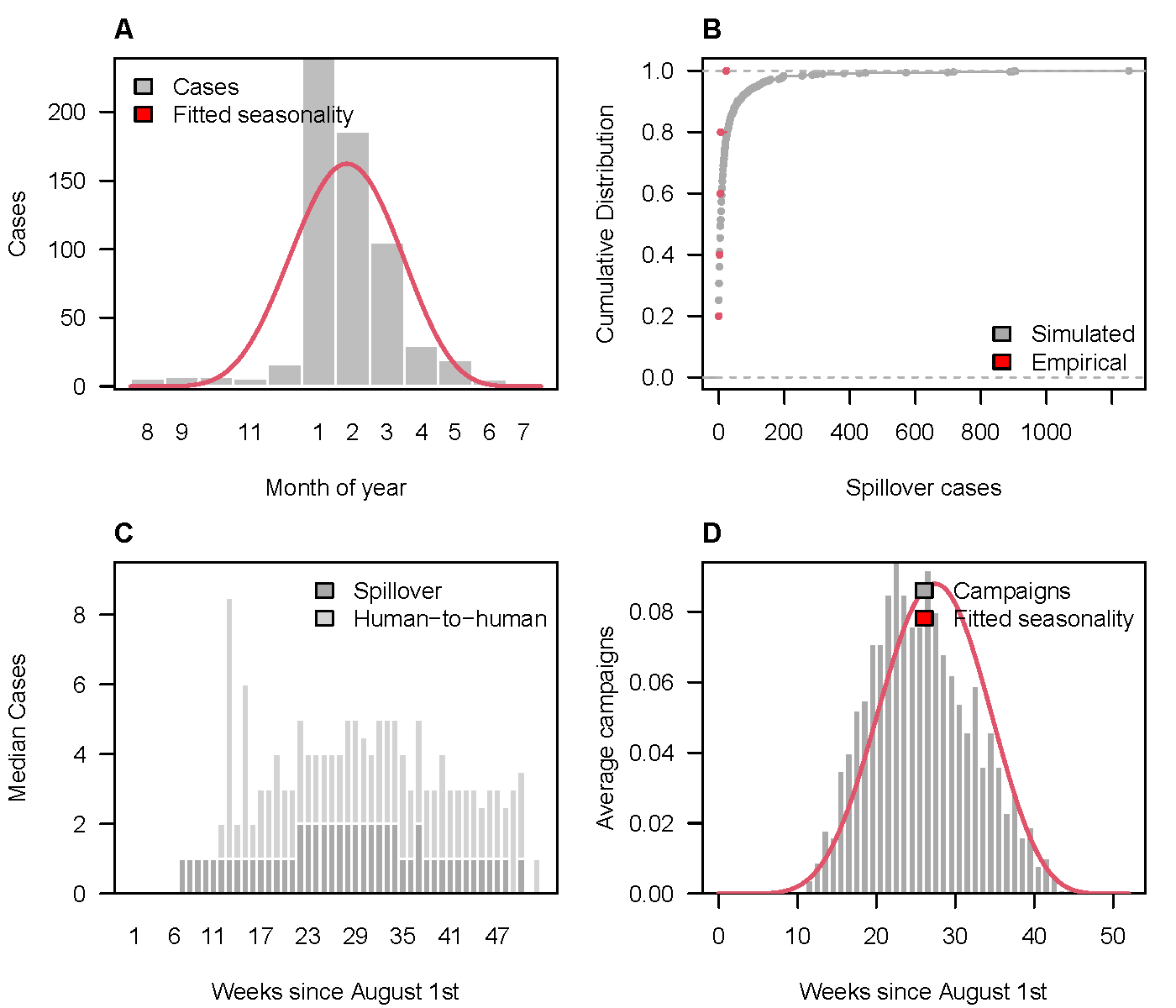
**

**Figure S3. Spillover and reactive vaccination patterns for Nipah virus (NiV).** (A) Observed weekly Nipah spillover cases (grey bars) and estimated seasonal spillover rate (red line). (B) Annual number of spillovers over the past 5 years (red) and cumulative distribution of simulated annual spillovers from 1000 replicates (grey). (C) Median weekly simulated spillover and human-to-human Nipah cases. (D) Average weekly number of reactive campaigns triggered via spillover detection compared to the estimated seasonal spillover rate (red line).

**
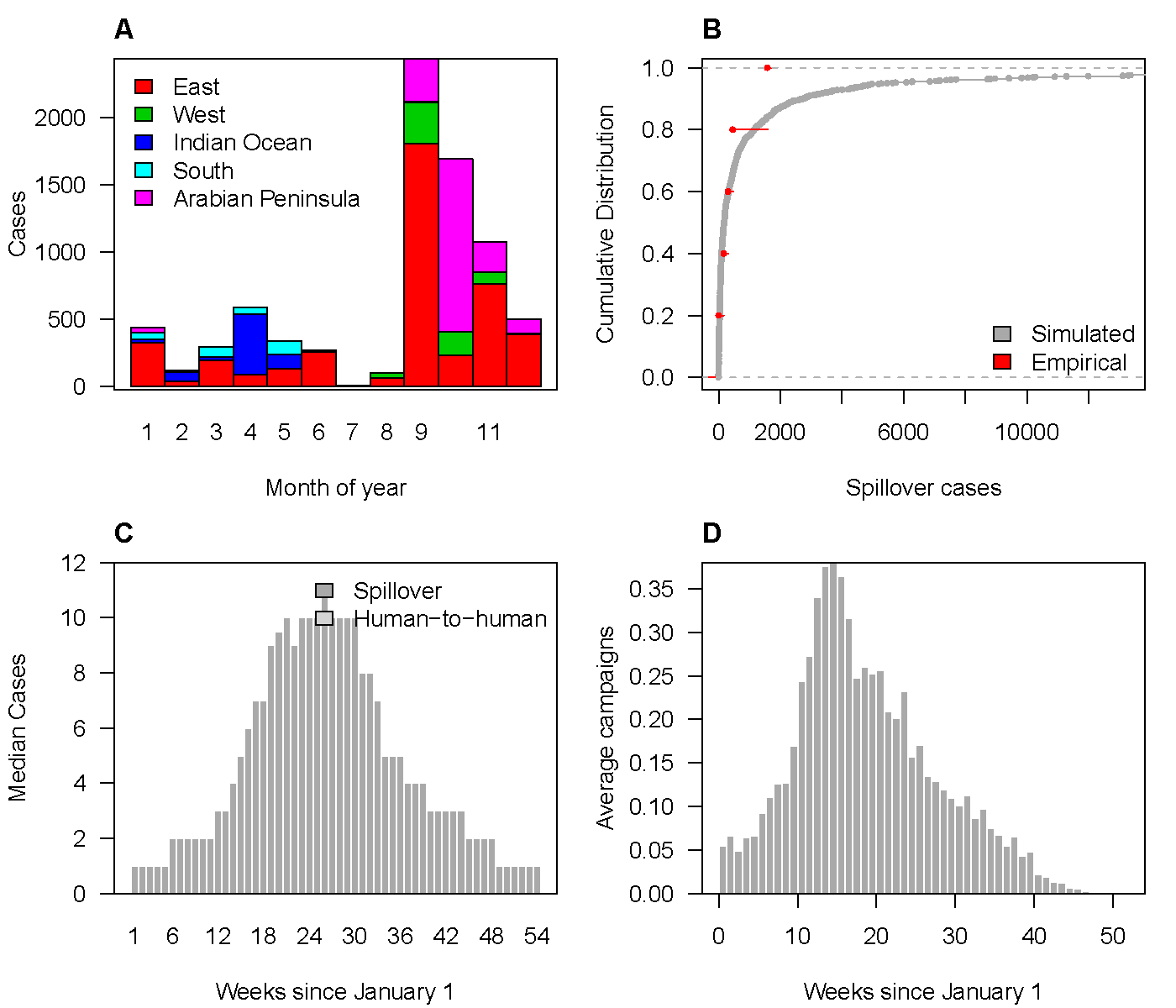
**

**Figure S4. Spillover and reactive vaccination patterns for Rift Valley fever virus (RVFV).** (A) Observed monthly RVF spillover cases by region. (B) Annual number of spillovers over the past 5 years (red) and cumulative distribution of simulated annual spillovers from 1000 replicates (grey). (C) Median weekly simulated spillover and human-to-human RVF cases. (D) Average weekly number of reactive campaigns triggered via spillover detection. Fitted seasonality is not shown for RVFV as it was for the other pathogens because seasonality was fit separately for each region.**SI Text.**

**1. Sensitivity analysis: Analysis of reactive vaccination scenarios**

In addition to our baseline reactive vaccination scenario, we examined the sensitivity of vaccination impact to varying different scenario parameters: the threshold number of cases needed to trigger a response, vaccination coverage in the general population, vaccination coverage of healthcare workers (HCWs), time from the threshold trigger to the start of vaccination, vaccine per exposure protection for one or two doses (PEP), and time delay from vaccination to protection (see Table 2 for default, low, and high parameter values). In addition, we examined the sensitivity of model results to the assumed or estimated value of R_0_ for each pathogen (SI Text section 2), and the impact of defining different catchment areas for vaccination (SI Text section 3). The results of the sensitivity analysis for MERS-CoV are summarized in the main text (Figure 6).

As expected, lowering the number of cases required to trigger a reactive vaccination campaign or increasing the percent of the population targeted for vaccination increased the number of vaccine regimens required for each pathogen (Figures S5-S8). These parameter values had a similar impact on the number of vaccine regimens needed to vaccinate HCWs (Figures S9-S12). Higher R_0_ values also increased the required number of vaccine regimens needed as higher human-to-human transmission increased the likelihood of the case threshold being exceeded. However, this impact was fairly small for LASV and RVFV due to their low R_0_ values (Figures S5,S8).

When vaccination impact was measured as the fraction of cases averted, the largest impact for each pathogen was achieved by lowering the threshold number of cases needed to trigger a reactive vaccination campaign (Figures 4A, S13A-15A). The second largest impact on the fraction of cases averted for each pathogen besides RVFV was achieved by decreasing the delay between a campaign being triggered and the start of vaccination (Figures 4A, S13A-S14A). For RVFV, the 2nd largest fraction of cases could be averted by increasing vaccination coverage in the general population or increasing vaccine PEP for a single dose vaccine (Figure S15A). Although lowering the response threshold maximized the fraction of cases averted for each pathogen, it did not maximize the number of cases averted per vaccine regimen administered, because lowering the threshold increased the number of vaccine campaigns and the required number of vaccine regimens (Figures S5-S8, 4B, S13B-S15B). For LASV, the largest per regimen impact was achieved by minimizing the delay prior to vaccination (Figure S13B). For NiV and RVFV, the largest per regimen impact was achieved by raising the response threshold (Figures S14B-S15B).

For each pathogen (besides RVFV, which is not associated with nosocomial transmission) vaccinating HCWs had a larger per regimen impact than vaccinating the general population (Figures 4C, S13C-S14C). Reducing the delay prior to vaccination and increasing vaccine PEP had the largest impact on the number of cases averted per HCW vaccinated (Figures 4C, S13C-S14C). The number of cases averted was slightly lower under our high-coverage of HCWs scenario because the high coverage of vaccination among HCWs was paired with low coverage among the general population (Figures 4A, S13A-S15A). However, this scenario would have achieved the highest number of cases averted per total number of vaccine regimens administered for LASV, MERS-CoV, and NiV.

**2. Sensitivity analysis: Impact of R_0_**

All R_0_ values and uncertainty ranges used in our analysis were either drawn from the literature or estimated from data (see Table 1 for parameter values and data sources). However, R_0_ estimates vary between studies and can also vary in space or time due to different environmental conditions or differences in human contact networks. Therefore, we also tested the sensitivity of our model results to lower and higher R_0_ values for each pathogen (for RVFV, the default R_0_=0, so only sensitivity to a higher value was examined). The number of total cases increased with R_0_ for each pathogen, with the largest sensitivity observed for MERS-CoV because the high estimate of R_0_ was close to 1 (Figure S16). There was also a large increase in the number of vaccine regimens required to vaccinate either the general population or HCWs for MERS-CoV at the higher R_0_ value, but the impact of R_0_ on the required number of vaccine regimens was minimal for the other pathogens (Figures S17-S18). As a result, there were minimal differences in the impact of vaccination under higher or lower R_0_ values for LASV, NiV, or RVFV (Figures S19-S22). Vaccination averted both a greater magnitude and a higher fraction of MERS cases as R_0_ increased (Figures S19-S20). In addition, the number of MERS cases averted per vaccine regimen administered to the general population or to HCWs also increased as R_0_ increased (Figures S21-S22). These results highlight the increasing potential effectiveness for reactive vaccination as a control strategy as R_0_ approaches 1 and larger outbreaks become more likely.

**3. Model limitations**

The goal of our analysis was to estimate vaccine stockpile needs and identify the most important determinants of success for reactive vaccination of zoonotic emerging pathogens. We modeled several different reactive vaccination strategies that are applicable to any zoonotic emerging pathogen, and tested this framework for four pathogens with differing epidemiologies. In addition, we explored the sensitivity of our results to different aspects of reactive vaccine deployment, such as the coverage level, deployment delays, and vaccine per exposure protection. However, there are some limitations to our approach that could affect these estimates.

First, we have a relatively poor understanding of the epidemiology of most emerging zoonotic pathogens, and data that could be used to try and elucidate the most important aspects of their epidemiologies is limited. Here we examined the impact of reactive vaccination for four pathogens with differing epidemiologies to try and capture how a range of epidemiological parameters (e.g., spillover rates, R_0_, etc.) affect vaccine stockpile requirements and the likely impact of vaccination. But there are still uncertainties surrounding the epidemiology of these pathogens that could affect the results of our analysis, such as the frequency of human-to-human transmission of MERS-CoV in community settings (68), or the route of NiV spillover to humans during recent outbreaks in India (69). In addition, because no vaccines have been licensed for these pathogens yet, we had to make assumptions about key vaccine parameters (e.g., number of doses, time between vaccination and protection, and per exposure protection), based on the current vaccine target product profiles (TPPs) for each pathogen. We also had to make assumptions about the baseline reactive vaccination campaign parameter estimates such as campaign response time and duration (and best-case and worst-case scenarios for our sensitivity analysis). Assessing vaccine stockpile needs for newly emerged pathogens will involve even more uncertainty as epidemiological knowledge is critically limited immediately following emergence, as was demonstrated following the 2019 emergence of SARS-CoV-2 (70,71). Our modeling approach can be applied to newly emerged zoonotic pathogens, but there will likely be a large amount of uncertainty regarding vaccine stockpile needs and where vaccination campaigns are most likely to occur.

A second, related, limitation, is that the modeling framework is intended to be applicable for a range of emerging zoonotic pathogens, and therefore cannot incorporate all of the specific epidemiological details that might affect vaccine demand or impact for a particular pathogen. For example, we assumed that the probability of transmission was uniform during the infectious period of a symptomatic individual. However, some diseases display a right-skewed infectiousness profile with the majority of transmission occurring relatively early during the infectious period. If the infectious period is highly right-skewed, with the median lower than the mean, our model may overestimate the impact of vaccination as fewer secondary cases would occur after the initiation of vaccine protection. On the other hand, if the infectious profile is left-skewed, with the majority of transmission occurring during the late stages of disease, then our model could underestimate vaccination impact as there would be more time to vaccinate HCWs following the identification of an index case.

Third, we only considered reported cases when estimating pathogen spillover rates and human-to-human transmission because undiagnosed or unreported infections would not trigger an outbreak response. For several of the pathogens considered, however, the majority of infections--and even symptomatic cases--go unreported. A frequently cited study estimated that LASV infects 100,000-300,000 and kills 5,000 people annually (72), and seroprevalence studies in several endemic areas indicate that spillover occurs much more frequently than reported (73–75). Seroprevalence surveys for RVFV and MERS-CoV also indicate that these pathogens cause many unreported infections in at least some subpopulations (76–78). Therefore, our estimate of reactive vaccination impact does not take into account the potential reduction in unobserved cases that would occur if at-risk populations were vaccinated. Improved surveillance could address this issue and would likely increase the frequency of reactive vaccination campaigns. This detection issue could also be partially addressed by adjusting the case threshold for outbreak response to account for the case detection probability, and then also adjusting vaccination impact to account for undetected infections.

Next, because the extent of community transmission for each of the study pathogens is poorly understood, we assumed that human-to-human transmission was limited to nosocomial settings. Although this could result in an underestimate of vaccine demand, our model simulations are consistent with epidemiological patterns observed to date (Figures S1-S4). We also assumed that all nosocomial transmission involved transmission from patient to HCWs or between HCWs and that there was no patient-to-patient or HCW-to-patient transmission. Therefore our estimates of the impact of vaccinating HCWs represents the upper-bound on the effectiveness of this strategy as instances of patient-to-patient transmission would not be prevented via this strategy.

Another simplifying assumption of our model is that cases in one catchment area do not lead to transmission or an outbreak outside of that catchment area. However, imported cases of these pathogens have been reported. A MERS-CoV outbreak in South Korea derived from a spillover event in the Middle East (59), a NiV outbreak in Singapore derived from a spillover event in Malaysia (79), and cases of Lassa fever have been imported to Europe (80). These types of events have been rare, and none of these documented events resulted in an outbreak larger than the range of those that we simulated. Furthermore, documented outbreaks involving pathogen spread to neighboring catchment areas are included in our datasets, and as such are to some extent captured in the current analysis. For example, our datasets include Lassa fever cases in Benin derived from an outbreak in the neighboring adm2 located in Nigeria and RVFV outbreaks within multiple catchment areas of Tanzania that likely resulted from the movement of livestock (11,78). Therefore, our model already implicitly incorporates the possibility of spread between-catchment areas, and although our model does not predict spillover cases occurring outside of each pathogen’s currently documented geographic distribution, the reactive vaccination strategies we examined should also be applicable for responding to imported cases and their associated outbreaks. However, because we do not incorporate the possibility that imported cases outside of a pathogen’s current geographical distribution might trigger an outbreak response, we may underestimate vaccine stockpile sizes.

Another limitation is that we had to make several simplifying assumptions regarding the implementation of the reactive vaccination campaigns. One such simplification was assuming that all vaccine doses (per regimen) were administered on the same day. This is likely an unrealistic assumption for mass vaccination campaigns, particularly those that cover large geographic areas. Relaxing this assumption would reduce the public health impact of reactive vaccination in the same manner that delays in the start of the vaccination campaign did in our analysis. However, lengthening the campaign beyond a single day would have a less detrimental effect than a comparable delay to the start of the campaign or a delay in time to protection following vaccination, because at least a fraction of the population would be vaccinated early on in the campaign window. Thus, our sensitivity analysis to delays in the campaign start date can be considered an upper bound on the impact of extending the length of the vaccination campaign. The median size of a single campaign targeting the general population ranged from 154,000 for LASV to 461,000 for NiV, suggesting that most campaigns could be completed in considerably less time than the 120-day delay that we considered in our sensitivity analysis.

Another simplification is that we did not consider any targeted vaccination strategies besides targeting healthcare workers to limit nosocomial outbreaks or ring vaccination around index cases. For the ring vaccination we calculated the number of index cases that would trigger a ring vaccination response, but we did not model the impact of this response. Besides these two strategies, there might be other targeted vaccination approaches that would require a smaller vaccine stockpile than targeting the general population while still producing a substantial public health impact. For example, in the case of RVFV, a potential vaccination strategy might include targeting high-risk groups such as veterinarians, butchers, and livestock holders. One reason we did not consider this strategy is because of the coarseness or absence of the data available on these professions. While veterinarians only constitute a small proportion of the at-risk population, their higher risk of acquiring infections could increase the impact of a campaign that targeted them for vaccination. This could also increase the safety of those that are often at the frontline of an outbreak response. Similar targeted strategies might be envisioned for camel workers in areas where MERS-CoV is endemic in livestock, or individuals who collect or consume date palm sap in India and Bangladesh (81,82).

Finally, spillover cases were distributed over catchment areas representing 2nd administrative districts (or hospitals within the 1st administrative units in our sensitivity analysis), irrespective of the urban/rural nature of the catchment area. This may result in an overestimation of the population at risk and thus the number of regimens needed. Simulating the spillover rates per 2nd administrative unit, instead of at the 1st administrative level, could improve the estimation of reactive vaccine demand. However, the adm2 location of spillover cases were not available most of the time.

**
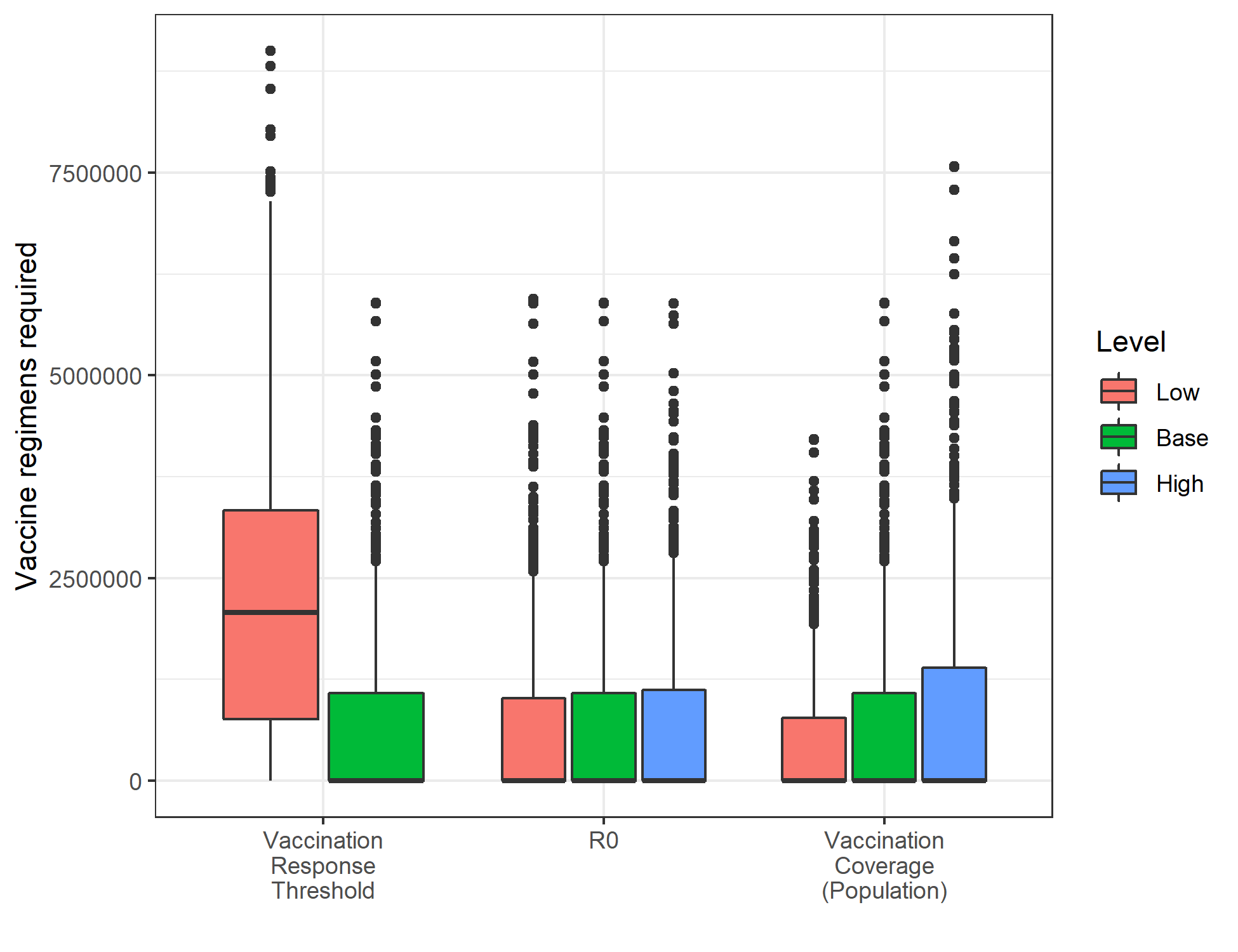
**

**Figure S5. Vaccine regimens required for Lassa fever virus (LASV).** The impact of varying several model parameters on the number of vaccine regimens required to meet reactive vaccination campaign targets. Base refers to the default scenario used in our main analysis. See Table 2 for specific parameter values.

**
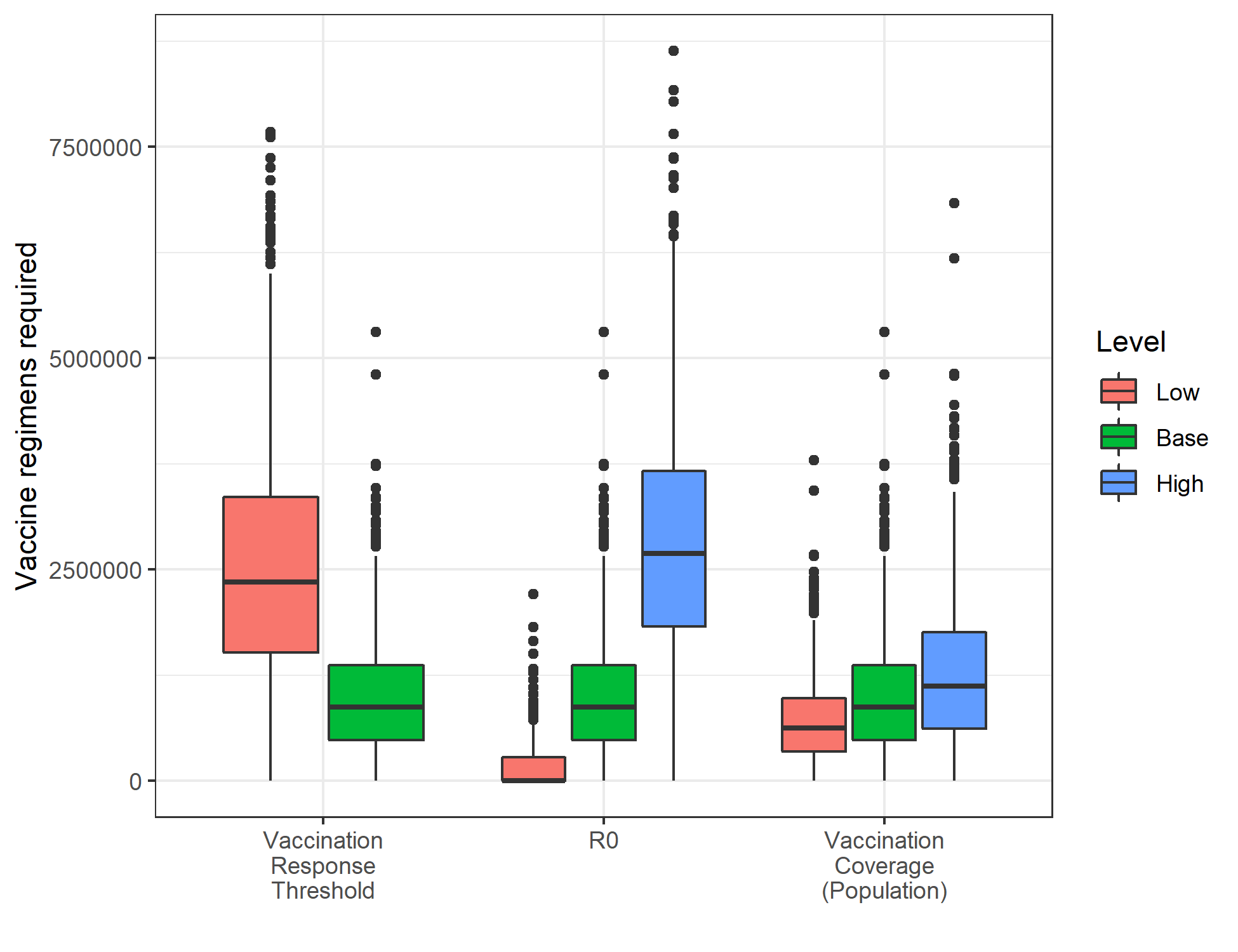
**

**Figure S6. Vaccine regimens required for Middle Eastern respiratory virus (MERS-CoV).** The impact of varying several model parameters on the number of vaccine regimens required to meet reactive vaccination campaign targets. Base refers to the default scenario used in our main analysis. See Table 2 for specific parameter values.

**
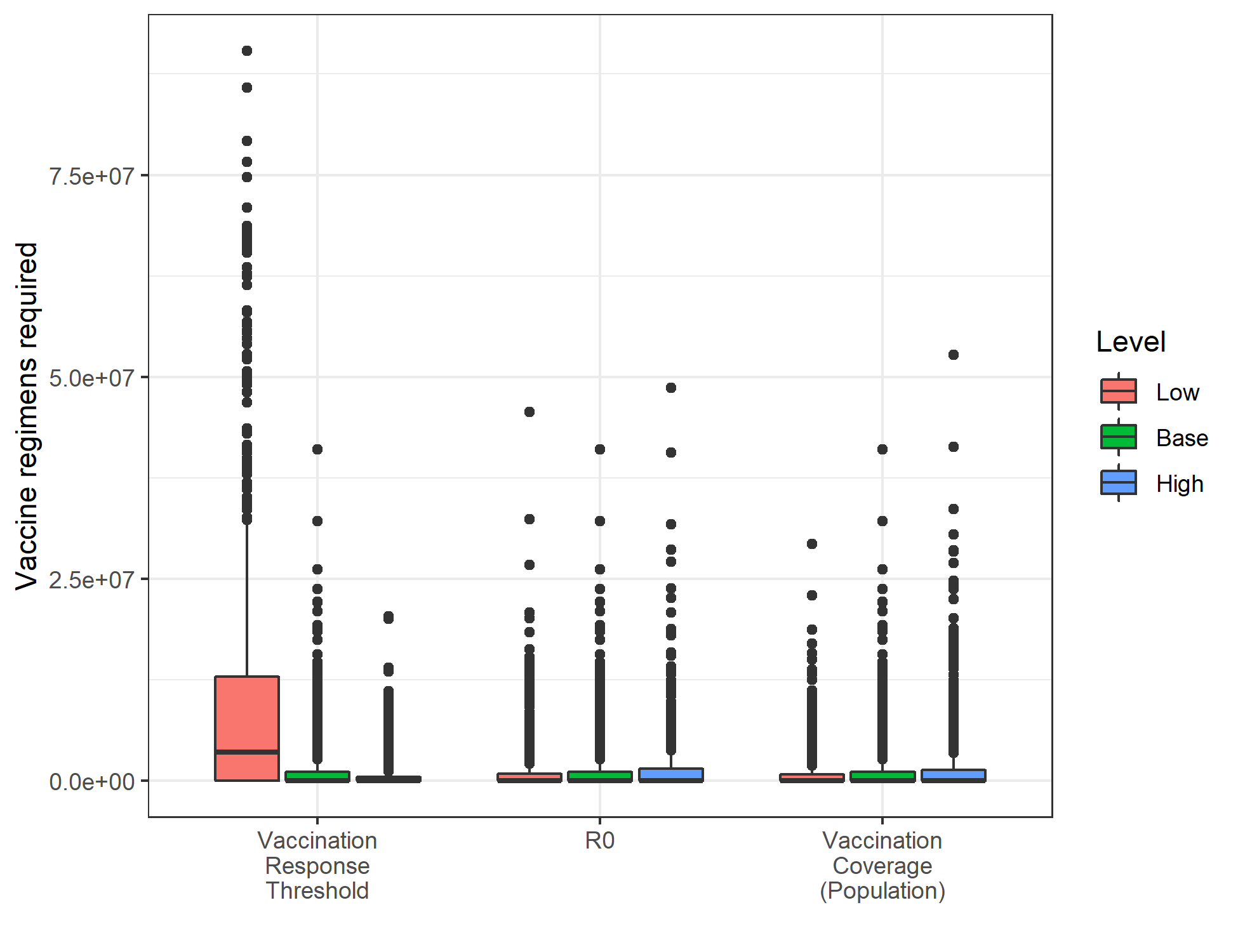
**

**Figure S7. Vaccine regimens required for Nipah virus (NiV).** The impact of varying several model parameters on the number of vaccine regimens required to meet reactive vaccination campaign targets. Base refers to the default scenario used in our main analysis. See Table 2 for specific parameter values.

**
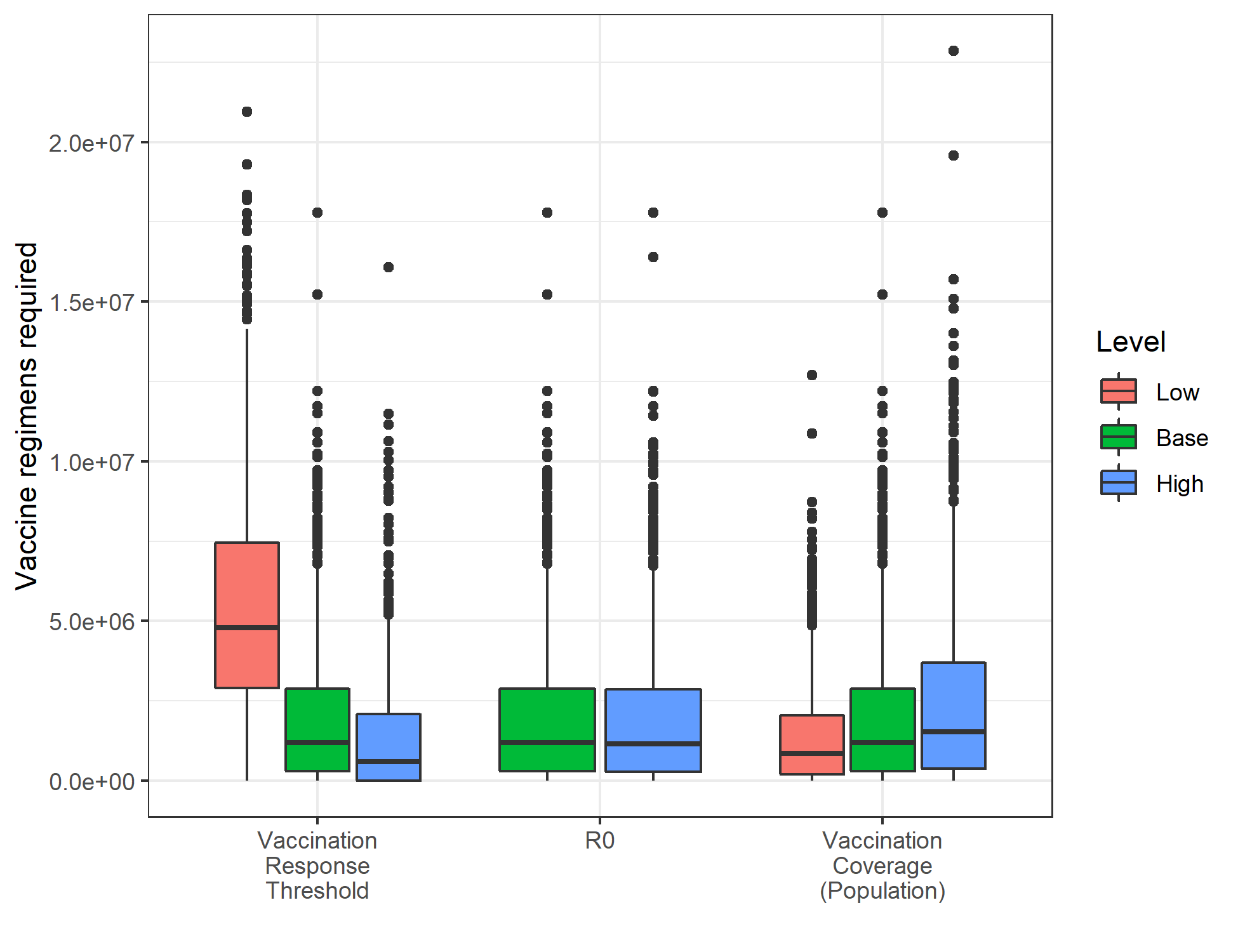
**

**Figure S8. Vaccine regimens required for Rift Valley fever virus (RVFV).** The impact of varying several model parameters on the number of vaccine regimens required to meet reactive vaccination campaign targets. Base refers to the default scenario used in our main analysis. See Table 2 for specific parameter values.

**
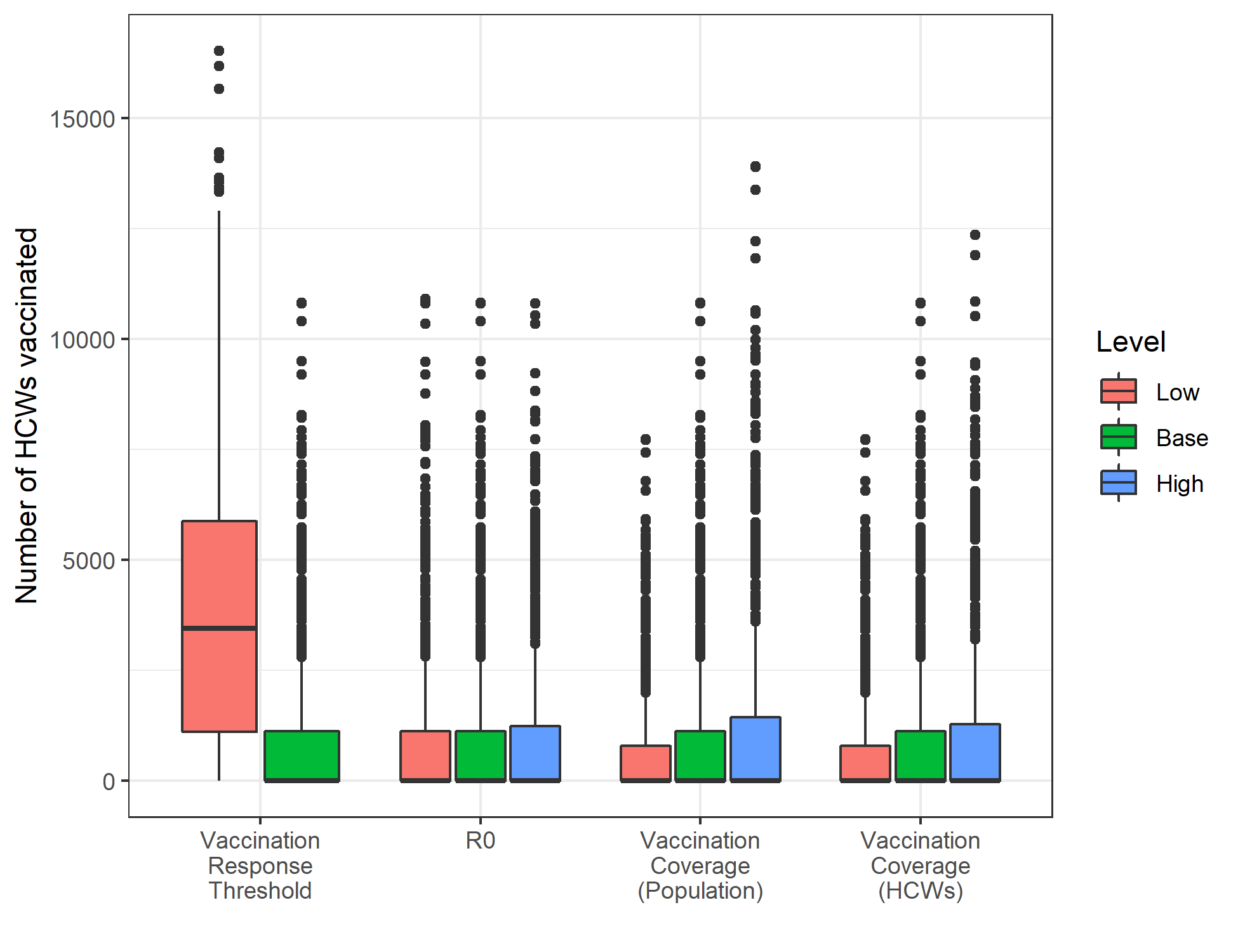
**

**Figure S9. Vaccine regimens required to vaccinate healthcare workers for Lassa fever virus (LASV).** The impact of varying several model parameters on the number of vaccine regimens required to meet reactive vaccination campaign targets among healthcare workers (HCWs). Base refers to the default scenario used in our main analysis. See Table 2 for specific parameter values.

**
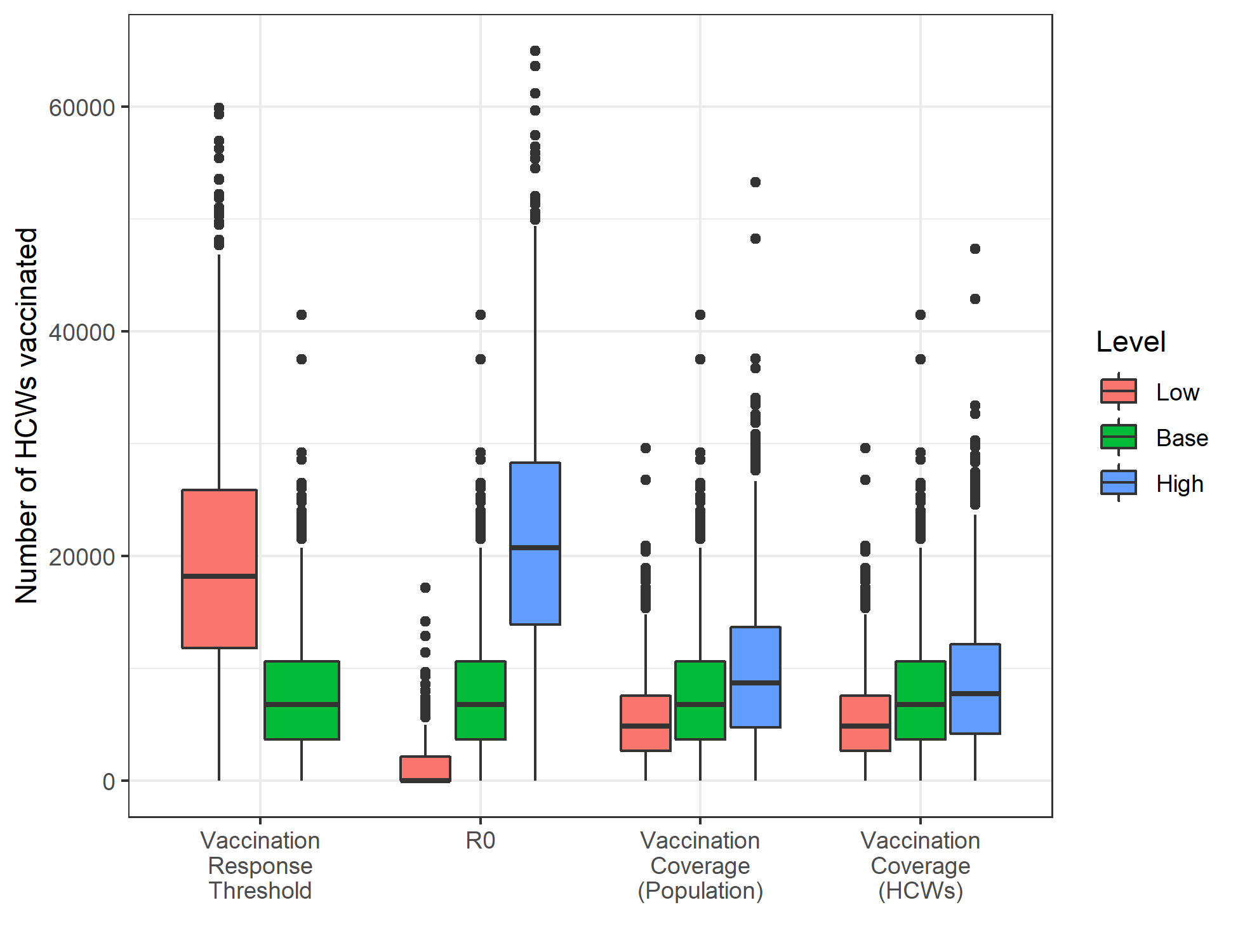
**

**Figure S10. Vaccine regimens required to vaccinate healthcare workers for Middle Eastern respiratory virus (MERS-CoV).** The impact of varying several model parameters on the number of vaccine regimens required to meet reactive vaccination campaign targets among healthcare workers (HCWs). Base refers to the default scenario used in our main analysis. See Table 2 for specific parameter values.

**
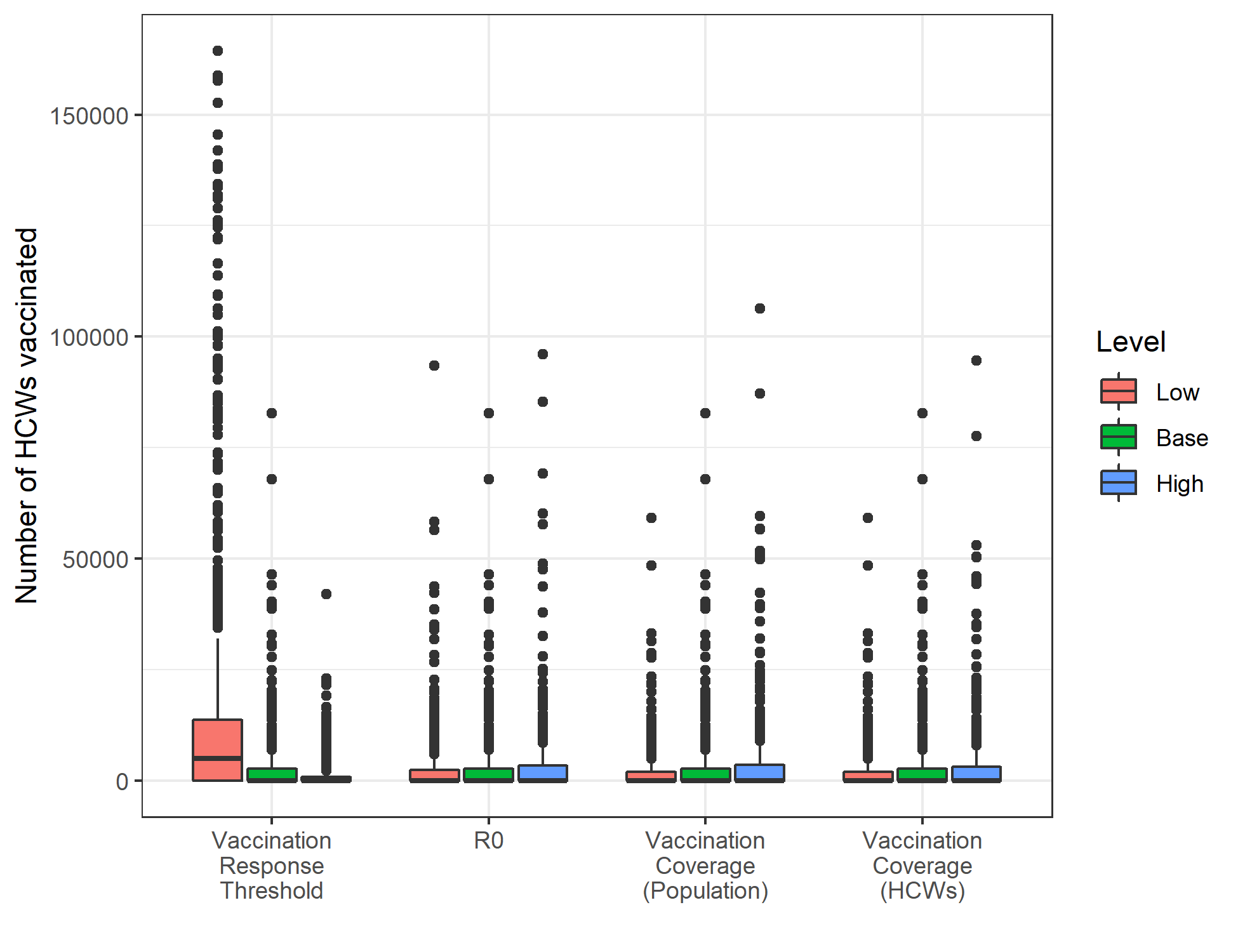
**

**Figure S11. Vaccine regimens required to vaccinate healthcare workers for Nipah virus (NiV).** The impact of varying several model parameters on the number of vaccine regimens required to meet reactive vaccination campaign targets among healthcare workers (HCWs). Base refers to the default scenario used in our main analysis. See Table 2 for specific parameter values.


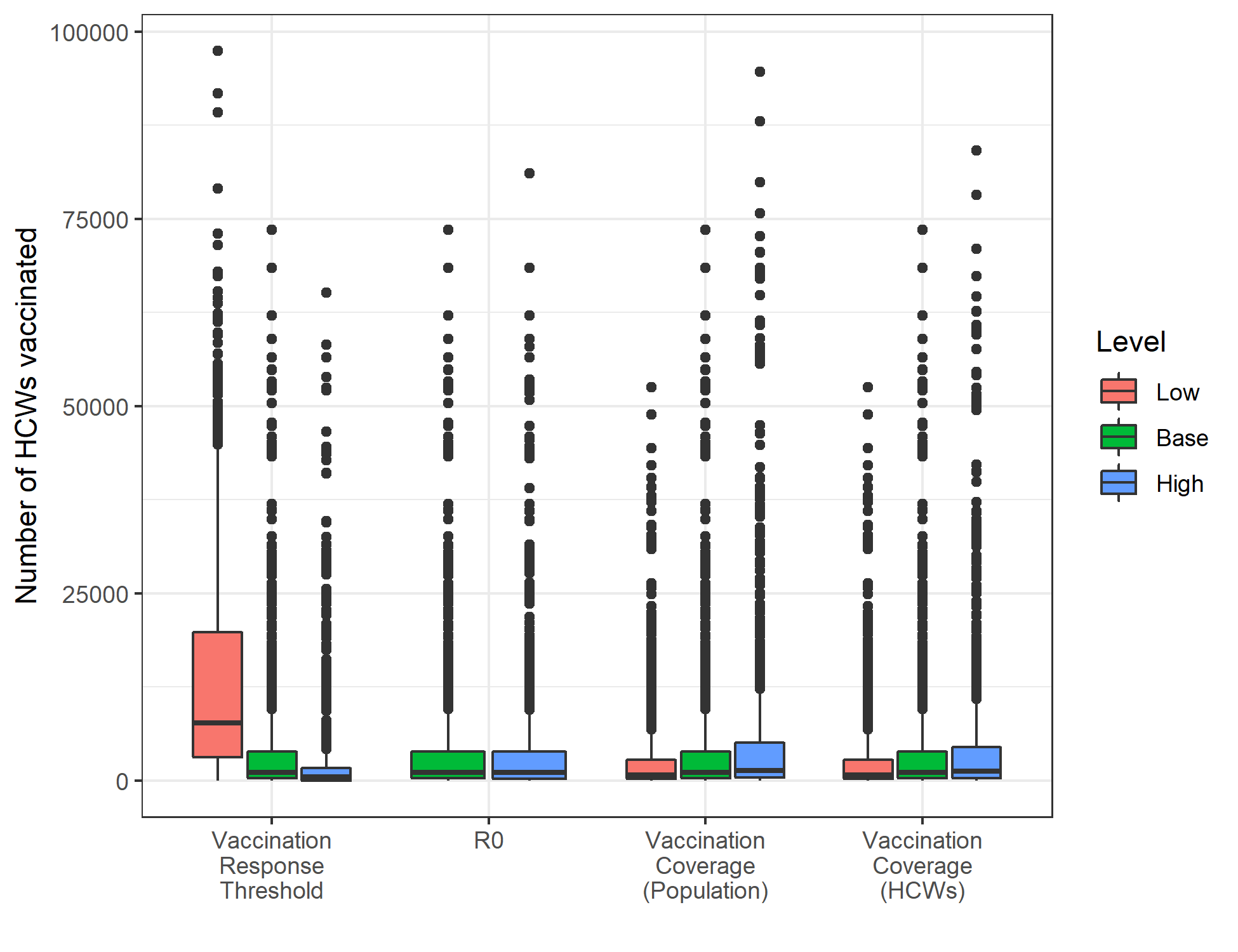


**Figure S12. Vaccine regimens required to vaccinate veterinarians for Rift Valley fever virus (RVFV).** The impact of varying several model parameters on the number of vaccine regimens required to meet reactive vaccination campaign targets among veterinarians (HCWs). Base refers to the default scenario used in our main analysis. See Table 2 for specific parameter values.

**
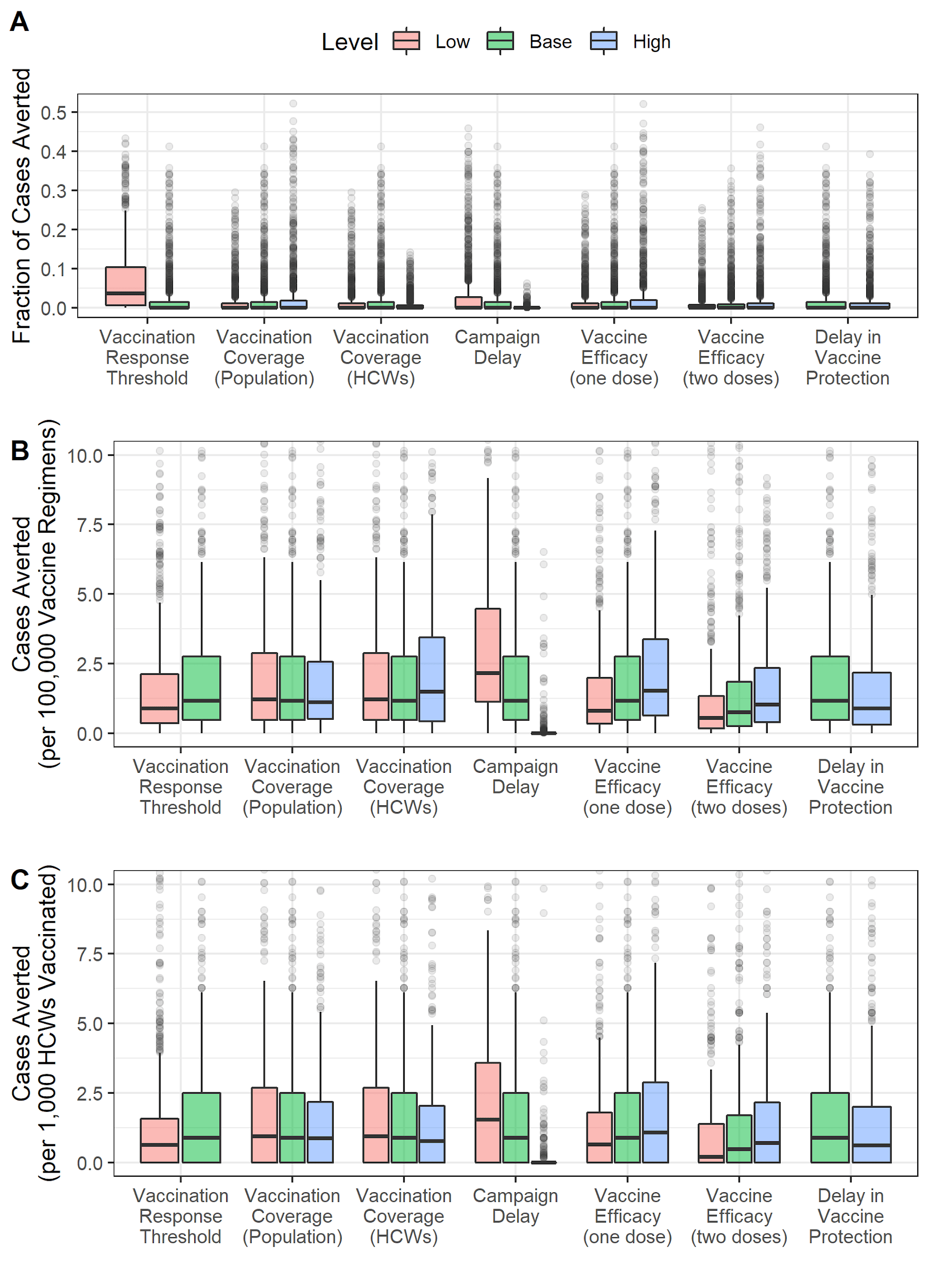
**

**Figure S13. Vaccination impact sensitivity analysis for LASV.** Sensitivity of vaccination impact for LASV to variation in different campaign parameters expressed as (A) fraction of cases averted, (B) cases averted per 100,000 vaccinated in the general population, and (C) cases averted per 1,000 health care workers (HCWs) vaccinated.

**
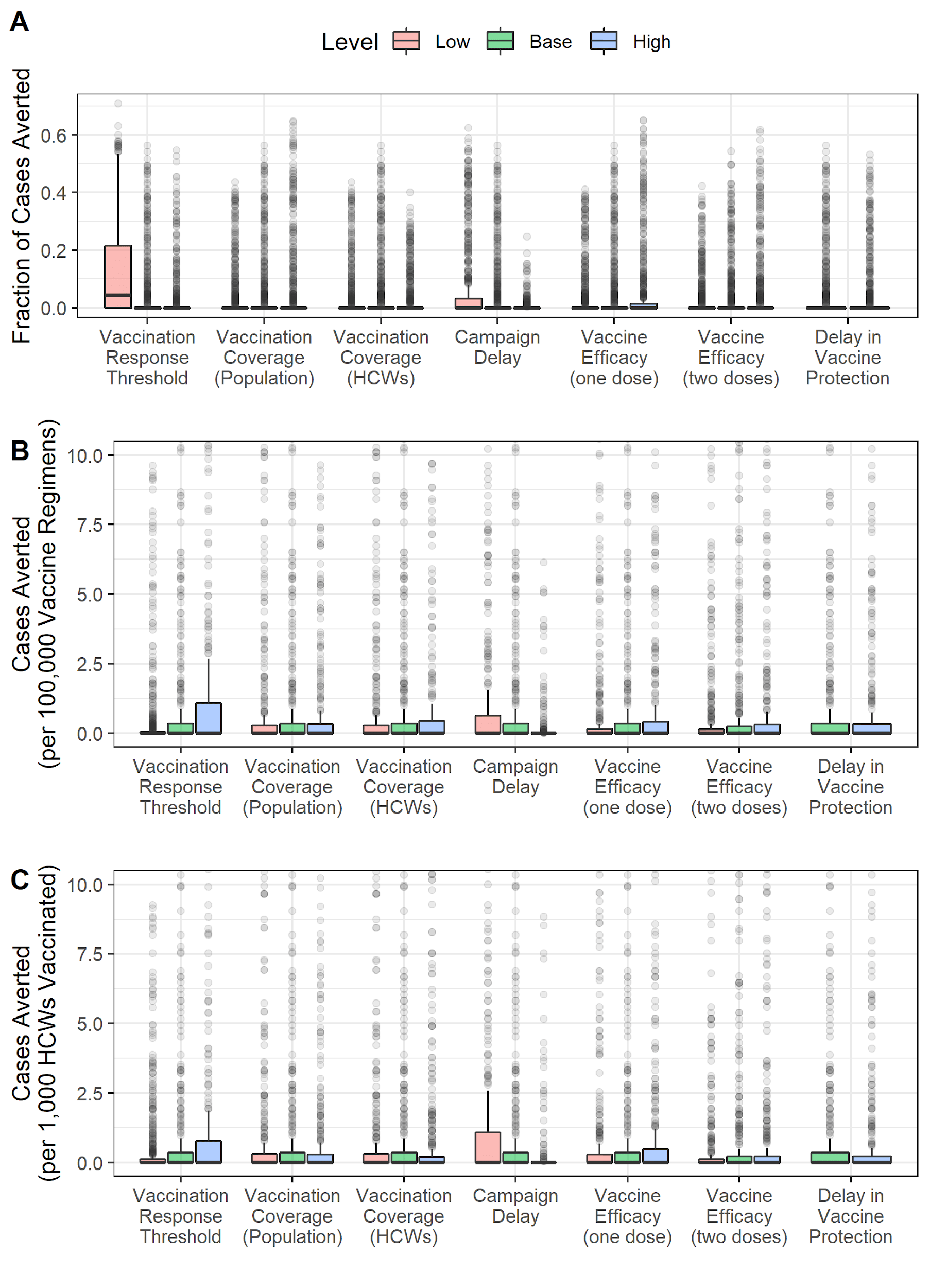
**

**Figure S14. Vaccination impact sensitivity analysis for NiV.** Sensitivity of vaccination impact for NiV to variation in different campaign parameters expressed as (A) fraction of cases averted, (B) cases averted per 100,000 vaccinated in the general population, and (C) cases averted per 1,000 health care workers (HCWs) vaccinated.

**
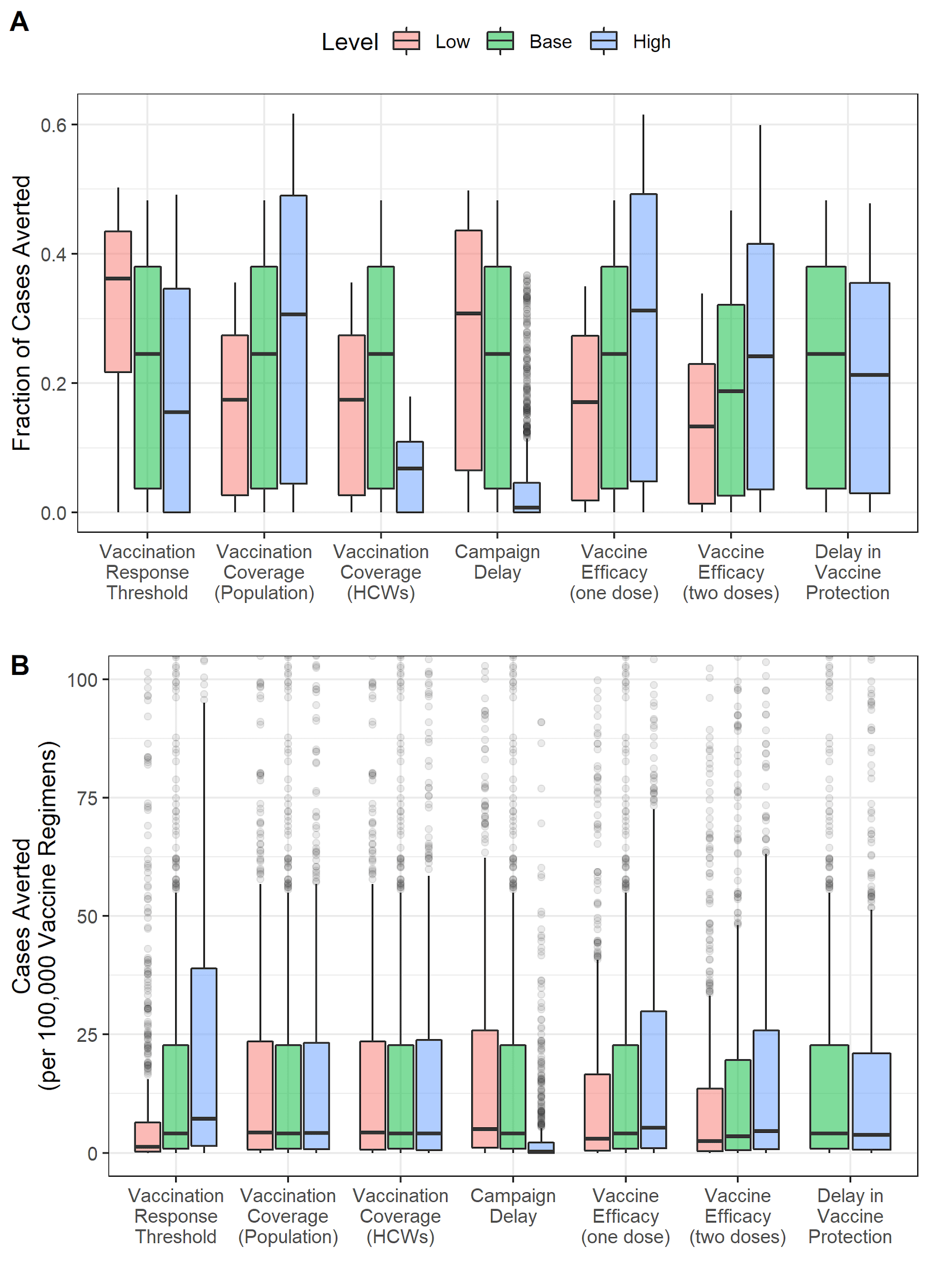
**

**Figure S15. Vaccination impact sensitivity analysis for RVFV.** Sensitivity of vaccination impact for RVFV to variation in different campaign parameters expressed as (A) fraction of cases averted, (B) cases averted per 100,000 vaccinated in the general population, and (C) cases averted per 1,000 health care workers (HCWs) vaccinated.

**
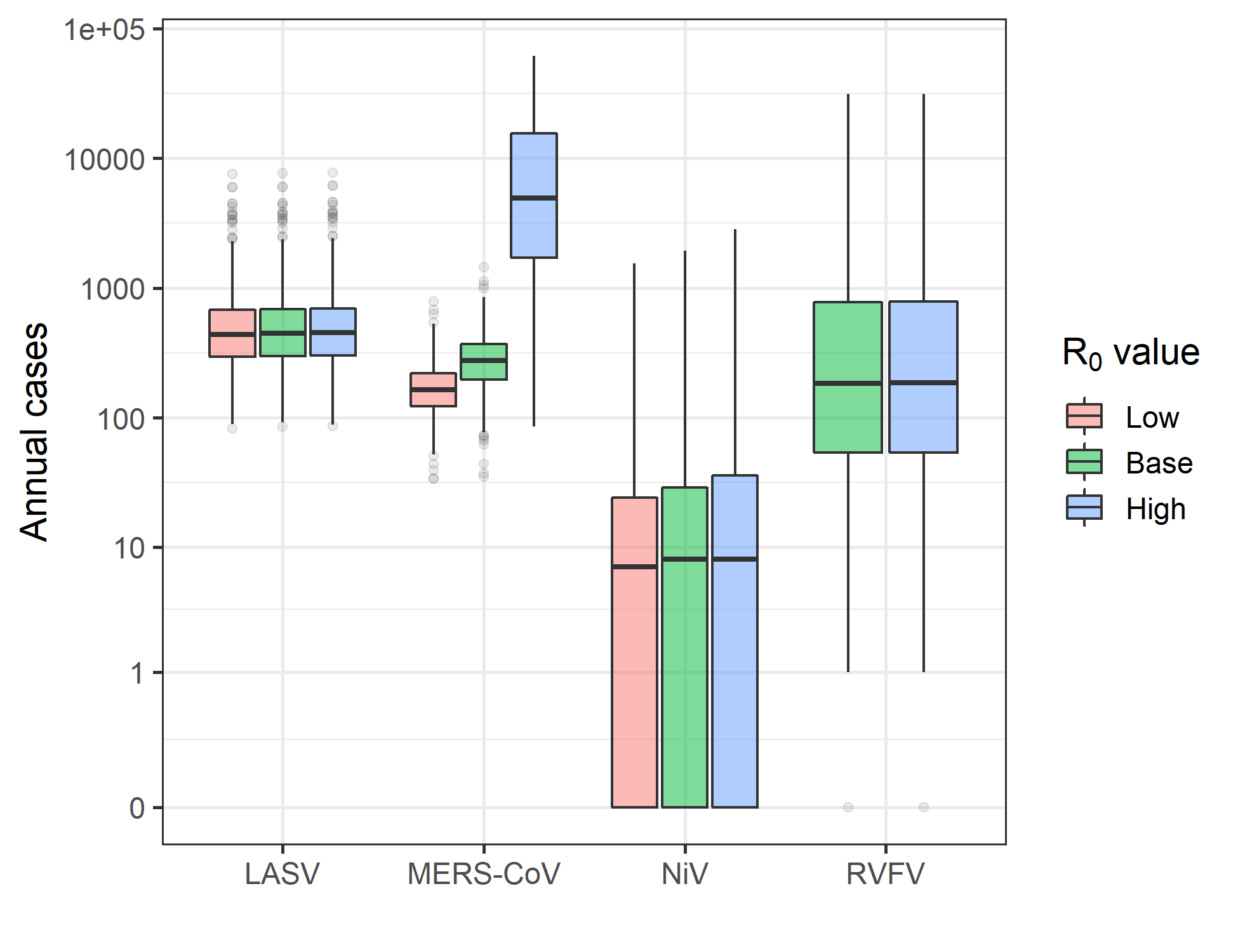
**

**Figure S16. Number of cases under different R_0_** **assumptions.**

**
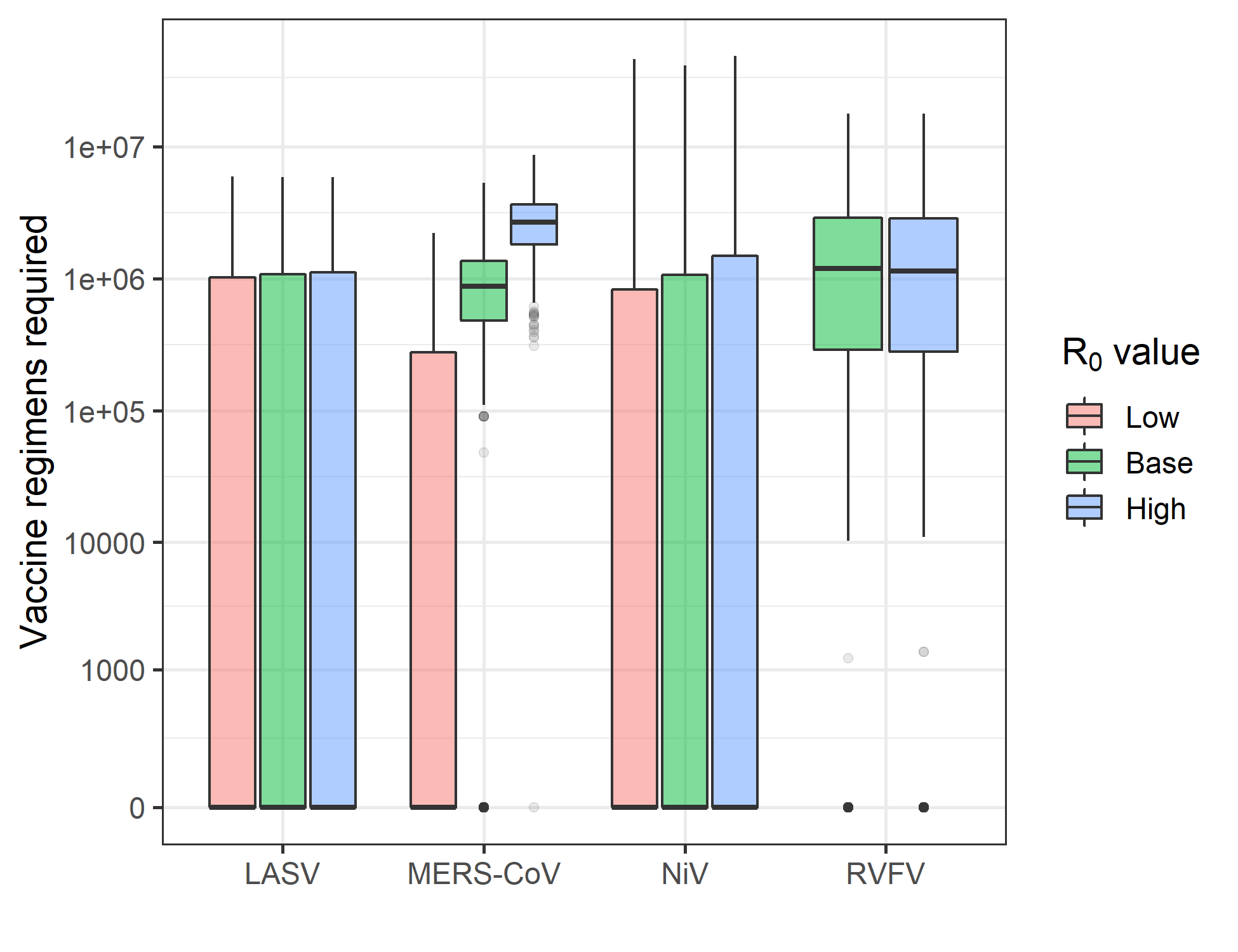
**

**Figure S17. Number of vaccine regimens required under different R_0_** **assumptions.**

**
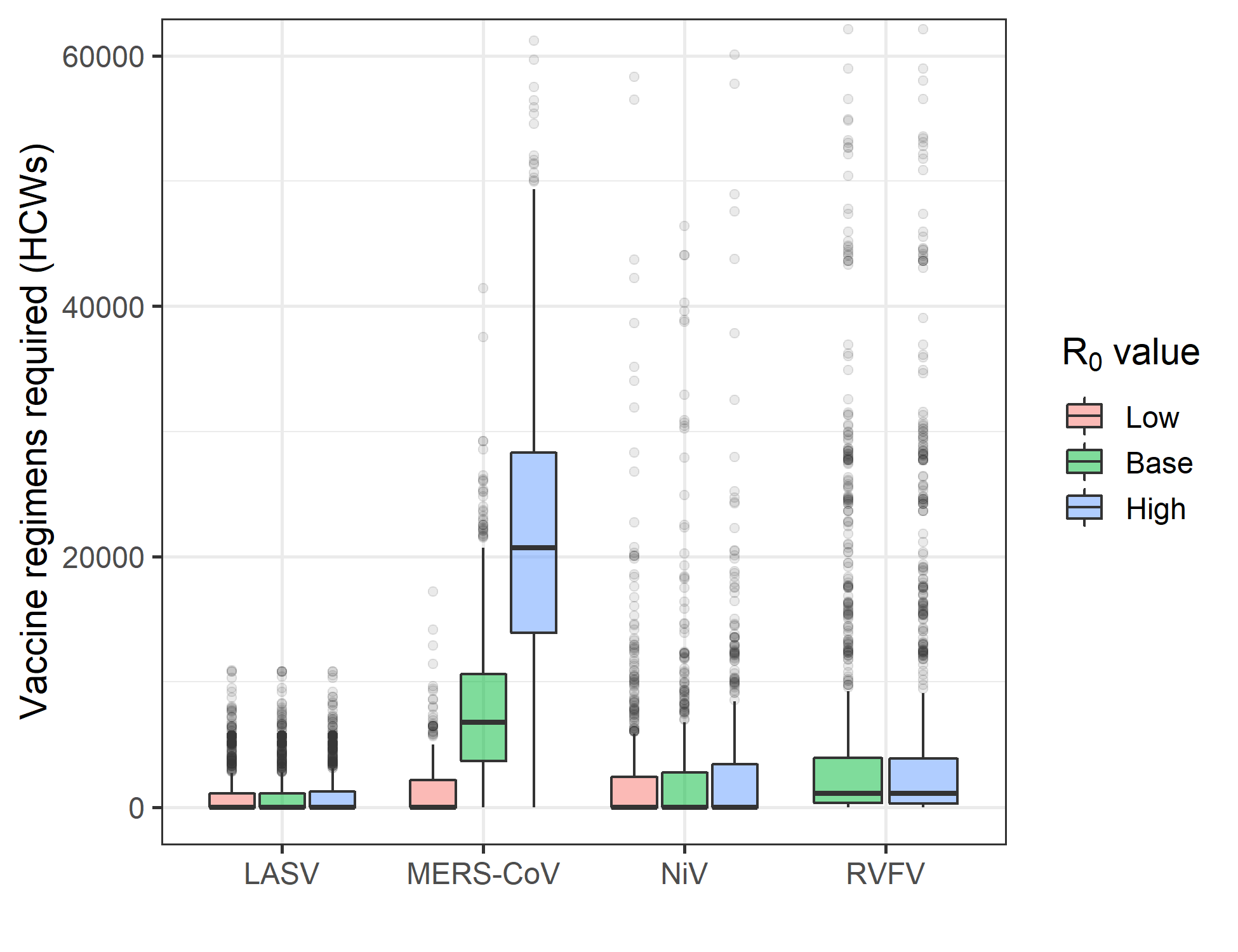
**

**Figure S18. Number of vaccine regimens required for healthcare workers (HCWs) under different R_0_** **assumptions.**

**
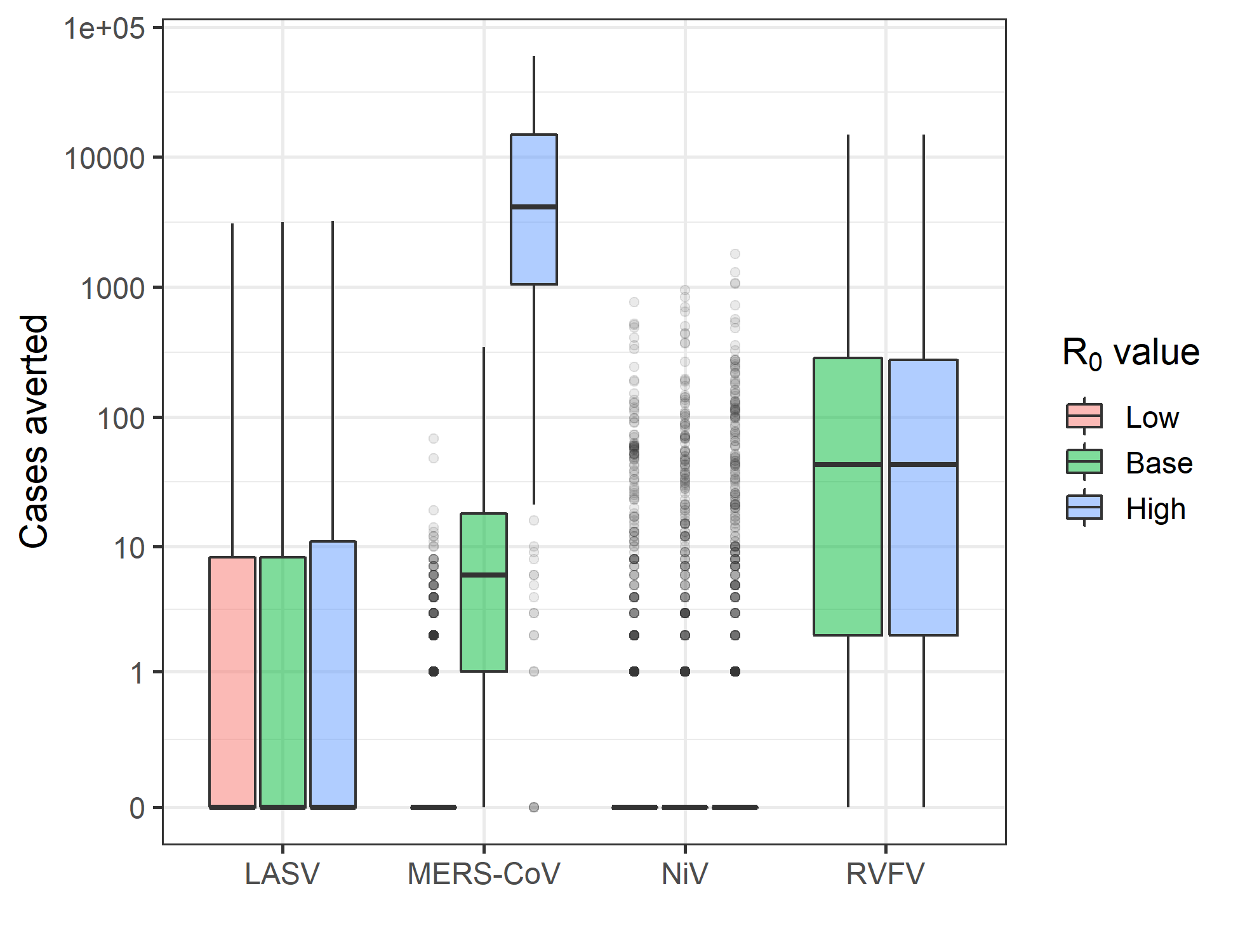
**

**Figure S19. Number of cases averted by vaccinating the general population under different R_0_** **assumptions.**

**
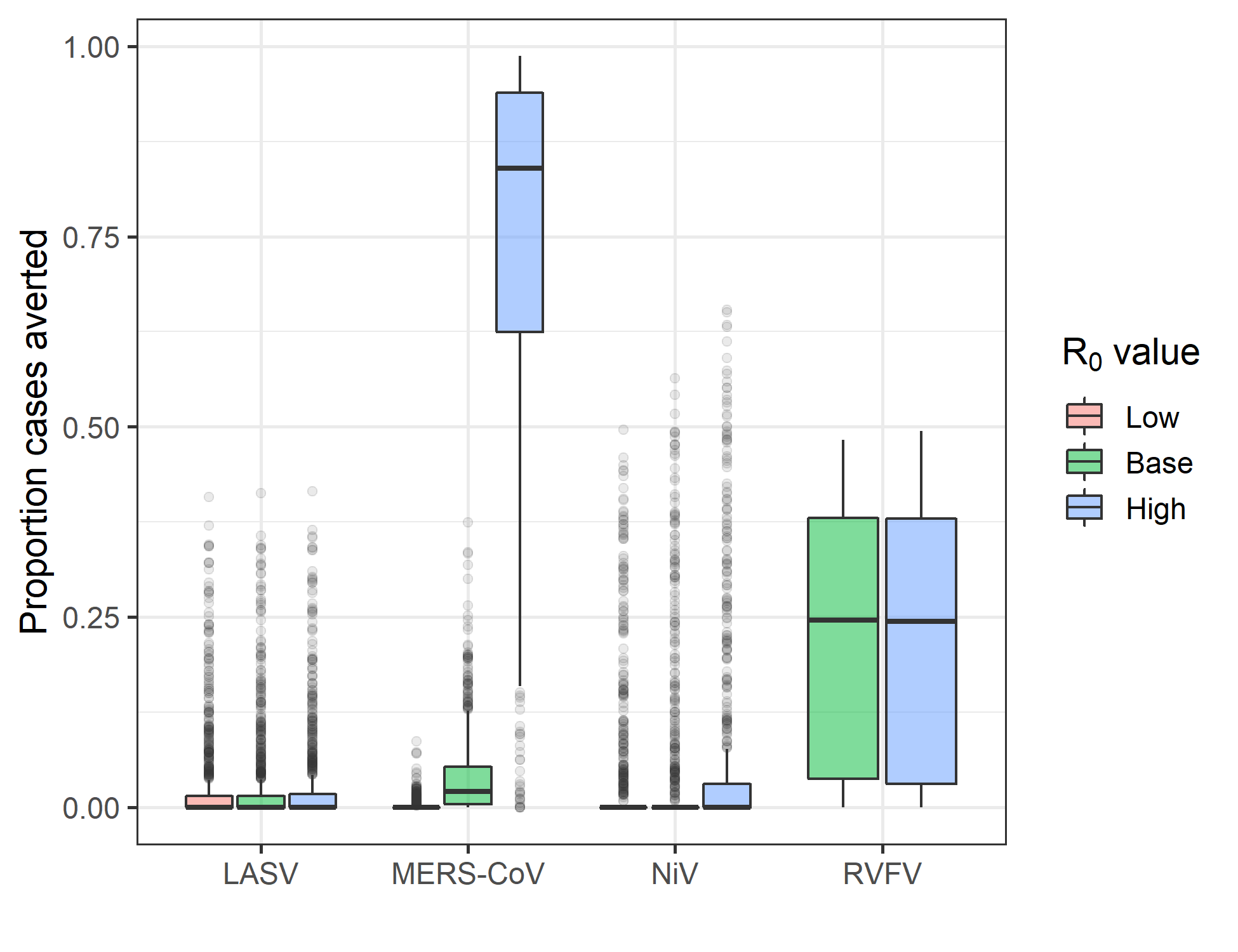
**

**Figure S20. Fraction of cases averted by vaccinating the general population under different R_0_** **assumptions.**

**
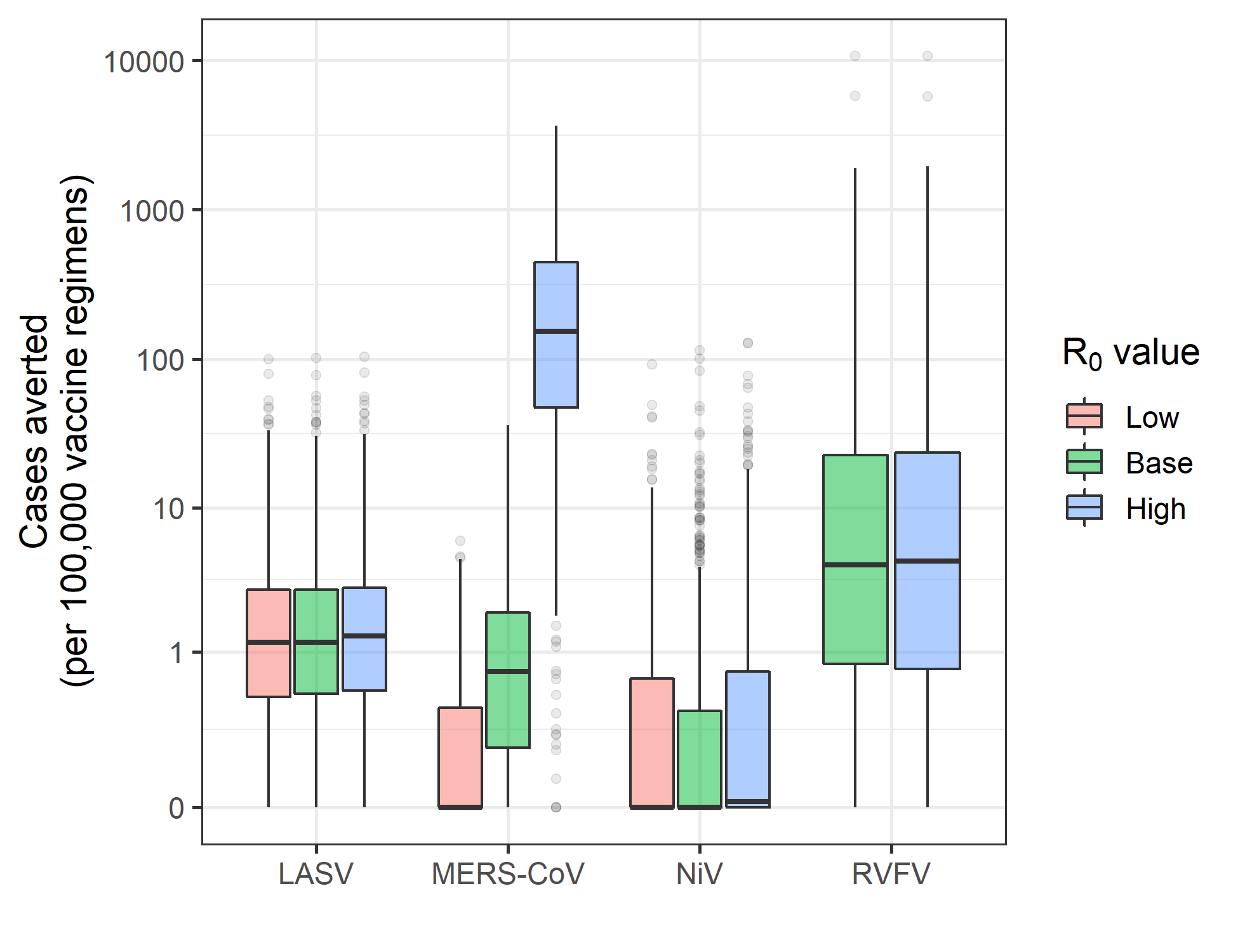
**

**Figure S21. Number of cases averted per vaccine regimen administered to the general population under different R_0_** **assumptions.**

**
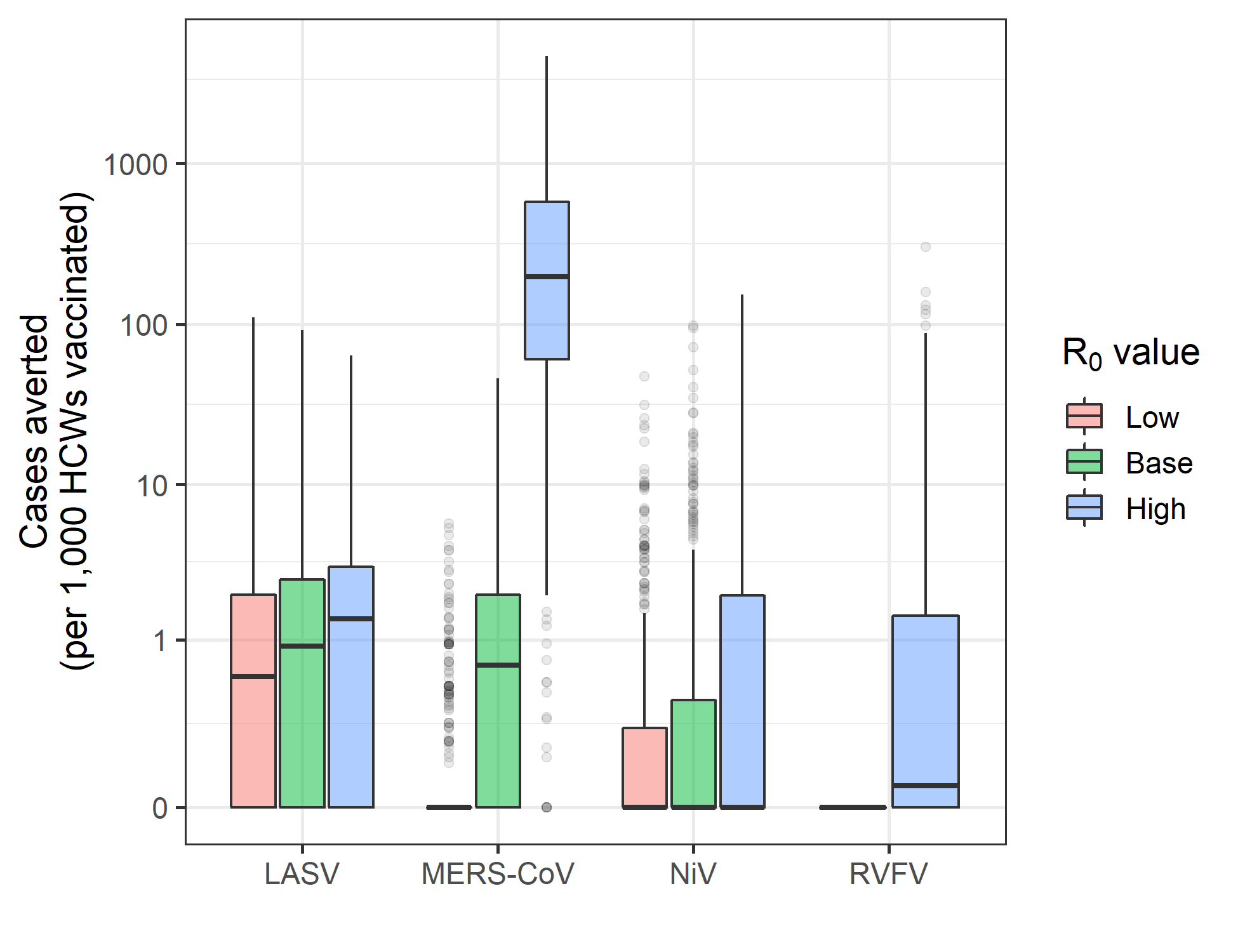
**

**Figure S22. Number of cases averted per vaccine regimen administered to healthcare workers (HCWs) under different R_0_** **assumptions.**

**4. Analysis of different spillover and vaccination catchment areas**

In our analysis we estimated spillover rates for each pathogen at the 1st administrative level (adm1). We then accounted for spatial clustering of cases below the adm1 level by associating each simulated case with a catchment area. In the main analysis, catchment areas were defined as the 2nd administrative units (adm2) within each adm1. For countries with no 2nd administrative level, hospitals within an adm1 were treated as catchment areas (hospitals within 10 km were combined into a single catchment area). This catchment area definition produced 1570 catchment areas for LASV, 767 for MERS-CoV, 5076 for NiV and 2126 for RVFV. Here we consider two alternative catchment area definitions: (1) treating all adm1 units as unique catchment areas, and (2) treating all hospitals within adm1 units as unique catchment areas (with hospitals <10km apart combined into a single catchment area). The adm1 catchment area definition resulted in 214 catchment areas for LASV, 82 for MERS-CoV, 375 for NiV and 343 for RVFV. The adm1 hospitals catchment area definition produced 1749 catchment areas for LASV, 3138 for MERS-CoV, 10799 for NiV and 4722 for RVFV. Therefore the adm1 catchment areas are larger than the adm2 catchment areas, while the adm1 hospitals catchment areas are generally smaller than the adm2 catchment areas. Because spillover rates were estimated at the adm1 level, spillovers within an adm1 unit were allocated via a multinomial distribution to all hospital catchment areas within that adm1 unit, with a probability of 1/(# of catchment areas within adm1).

The number of spillover cases remained the same under the different catchment area definitions, but the frequency, timing, and location of reactive vaccination campaigns were shifted. Reactive vaccination campaigns tended to be triggered sooner during the transmission season for adm1 catchment areas because these catchment areas covered a broader area and larger population (Figures S23-S26). There were only minor differences in the timing of vaccination campaigns between the adm2 and adm1 hospitals catchment areas (Figures S1-S4, S27-S30). The geographic distribution of spillovers was less clustered for the larger adm1 catchment areas, but more finely distributed and clustered for the adm1 hospital catchment areas, particularly in countries like South Africa and Madagascar with large 1st-level administrative regions (Figures 2A, S31A-S32A). A similar geographic pattern was observed for the location of reactive vaccination campaigns (Figures 2B, S31B-S32B).

The total number of human-to-human cases did not differ by catchment area definition for any of the pathogens (Figures 1A, S33A-S34A). The median number of reactive vaccination campaigns was lower using adm1 catchment areas than adm2 catchment areas for MERS-CoV (3; 95% PrI: 1-6 vs. 4; 95% PrI: 0-11) and RVFV (3; 95% PrI:0-6 vs. 5; 95% PrI: 0-20) (Figures 1B, S33B). In contrast, the median number of reactive vaccination campaigns were higher using adm1 catchment areas for LASV (3; 95% PrI: 1-6 vs. 0; 95% PrI: 0-20) and NiV (1; 95% PrI: 0-3 vs. 0; 95% PrI: 0-8). The frequency of years with no reactive vaccination campaigns was lower using adm1 catchment areas compared to adm2 or adm1 hospital catchment areas for all four pathogens. Both the median number of reactive vaccination campaigns and the frequency of years with no campaigns were similar using adm1 hospital catchment areas compared to the baseline adm2 catchment areas, although the frequency of years with no campaigns was slightly higher for the adm1 hospital catchment areas for all four pathogens (Figures 1B, 34B).

The number of vaccine regimens required to cover the general population were significantly higher using adm1 catchment areas versus adm2 or adm1 hospital catchment areas for (Figures 1C, S33C-34C). For MERS-CoV, the median required number of regimens increased from 286,259 (95% PrI: 0-855,099) for adm1 hospital catchment areas, to 1,242,922 (95% PrI: 0-4,062,010) for adm2 catchment areas, and 14,303,325 (95% PrI: 1,416,969) for adm1 catchment areas. For LASV, the median required number of regimens increased from 0 (95% PrI: 0-4,452,966) for adm1 hospital catchment areas and 0 (95% PrI: 0-5,184,360) for adm2 catchment areas, to 11,603,802 (95% PrI: 2,953,628-25,277,594) for adm1 catchment areas. The number of vaccine regimens needed to vaccinate healthcare workers (HCWs) was also lowest for adm1 hospital catchment areas and highest for adm1 catchment areas (Figures 1C, S33C-S34C).

The total number of cases averted via vaccination for each pathogen was also lowest using adm1 hospital catchment areas and highest using adm1 catchment areas (Figures 1D, S33D-S34D). For MERS, the median number of cases averted increased from 2 (95% PrI: 0-60) for adm1 hospital catchment areas, to 6 (95% PrI: 0-83) for adm2 catchment areas, and 77 (95% PrI: 0-342) for adm1 catchment areas. For Lassa fever, the median number of cases averted increased from 0 (95% PrI: 0-306) for adm1 hospital catchment areas and 0 (95% PrI: 0-357) for adm2 catchment areas, to 101 (95% PrI: 3-771) for adm1 catchment areas. For RVF, the median number of cases averted increased from 29 (95% PrI: 0-3,525) for adm1 hospital catchment areas, to 43 (95% PrI: 0-5,826) for adm2 catchment areas, and 66 (95% PrI: 0-2,451) for adm1 catchment areas. For Nipah, the median number of cases averted was 0 for each catchment area definition, but the mean was highest for adm1 catchment areas. The total number of cases averted via vaccination of HCWs was also lowest using adm1 hospital catchment areas and highest using adm1 catchment areas (Figures 1D, S33D-S34D). For example, for MERS, the median number of nosocomial cases averted increased from 1 (95% PrI: 0-60) for adm1 hospital catchment areas, to 4 (95% PrI: 0-77) for adm2 catchment areas, and 55 (95% PrI: 0-259) for adm1 catchment areas.

Although the number of cases averted via reactive vaccination was highest using adm1 catchment areas, the number of cases averted per vaccine regimen administered was not necessarily the highest under this scenario because the number of regimens required was also higher using adm1 catchment areas. For MERS, the highest per regimen impact was achieved using adm1 hospital catchment areas where a median of 0.75 (95% PrI: 0-18.10) cases were averted per 100,000 vaccine regimens administered. In comparison, a median of 0.58 (95% PrI: 0.02-2.58) cases were averted per 100,000 vaccine regimens administered in adm1 hospital catchment areas, and 0.49 (95% PrI: 0-5.21) cases were averted per 100,000 vaccine regimens administered in adm2 catchment areas. The highest per regimen impact for RVF was also achieved using adm1 hospital catchment areas, with a median of 3.18 cases averted per 100,000 vaccine regimens administered versus 2.86 (95% PrI: 0-349.78) using adm2 catchment areas or 1.69 (95% PrI: 0-68.42) using adm1 catchment areas. For Lassa fever the per regimen impact was relatively consistent across different catchment areas, and for Nipah the median impact per 100,000 vaccine regimens administered was 0 for adm2 or adm1 hospital catchment areas and 0.01 (95% PrI: 0-11.17) for adm1 catchment areas. For MERS and Lassa fever, the largest impact of vaccinating HCWs as measured on a per-regimen-administered basis, was also achieved using adm1 hospital catchment areas. The per-regimen impact of vaccinating HCWs was minimal for Nipah, just as it was for vaccinating the general population (although the estimated per-regimen impact of vaccinating HCWs was higher than the impact of vaccinating the general population).


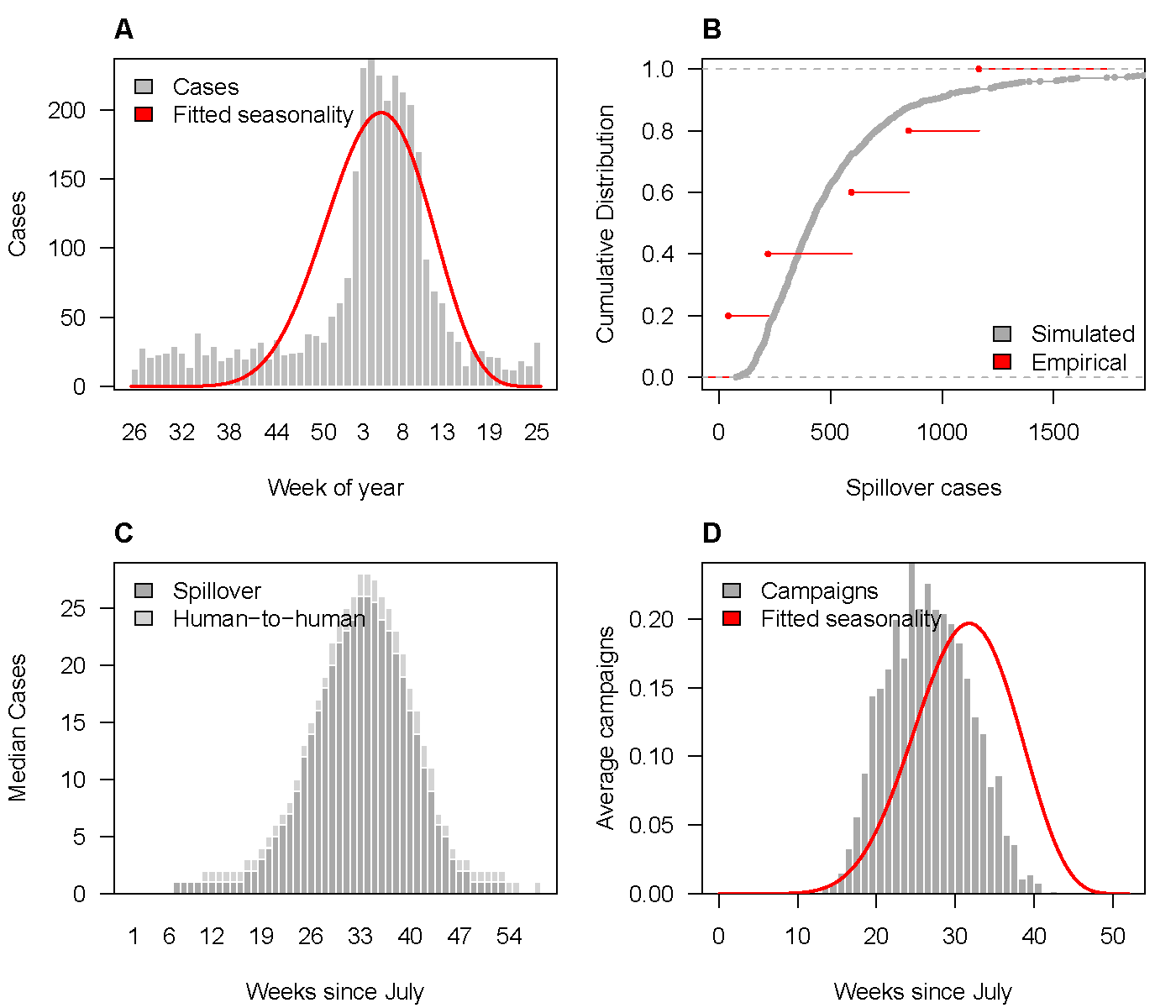


**Figure S23. Spillover and reactive vaccination patterns for Lassa fever virus (LASV) within adm1 catchment areas.** (A) Observed weekly Lassa fever spillover cases (grey bars) and estimated seasonal spillover rate (red line). (B) Annual number of spillovers over the past 5 years (red) and cumulative distribution of simulated annual spillovers from 1000 replicates (grey). (C) Median weekly simulated spillover and human-to-human Lassa fever cases. (D) Average weekly number of reactive campaigns triggered via spillover detection compared to the estimated seasonal spillover rate (red line).

**
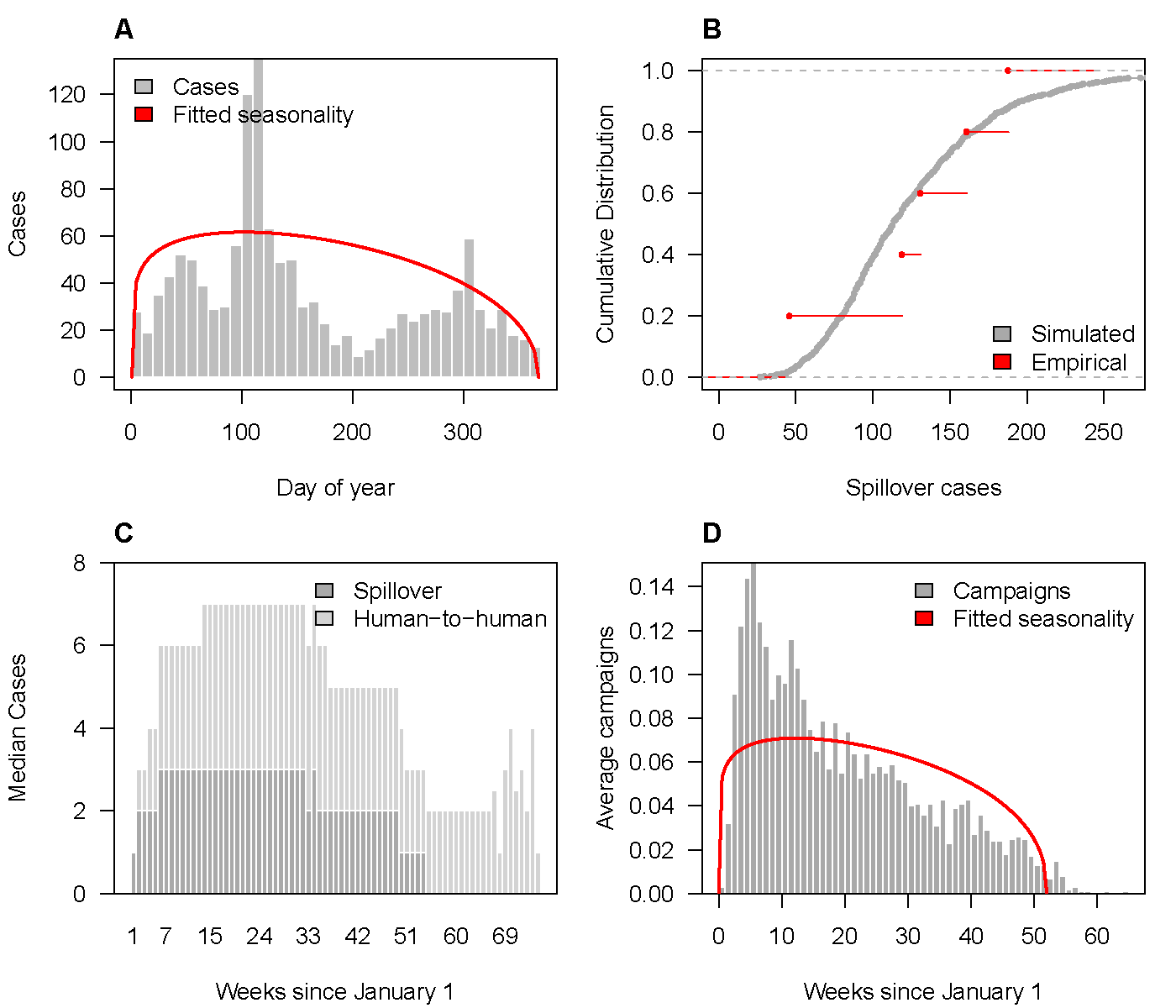
**

**Figure S24. Spillover and reactive vaccination patterns for Middle Eastern respiratory virus (MERS-CoV) within adm1 catchment areas.** (A) Observed weekly MERS spillover cases (grey bars) and estimated seasonal spillover rate (red line). (B) Annual number of spillovers over the past 5 years (red) and cumulative distribution of simulated annual spillovers from 1000 replicates (grey). (C) Median weekly simulated spillover and human-to-human MERS cases. (D) Average weekly number of reactive campaigns triggered via spillover detection compared to the estimated seasonal spillover rate (red line).

**
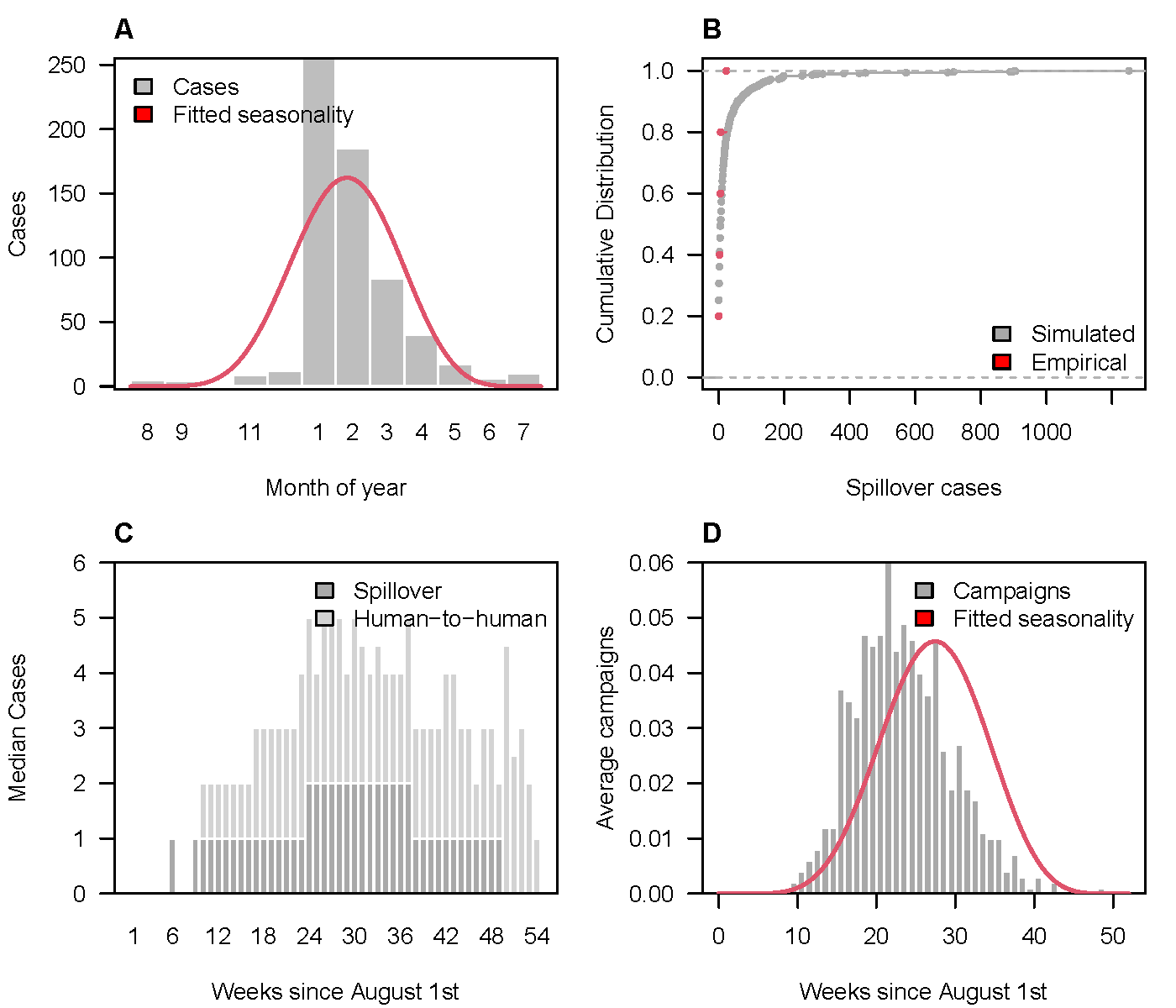
**

**Figure S25. Spillover and reactive vaccination patterns for Nipah virus (NiV) within adm1 catchment areas.** (A) Observed weekly Nipah spillover cases (grey bars) and estimated seasonal spillover rate (red line). (B) Annual number of spillovers over the past 5 years (red) and cumulative distribution of simulated annual spillovers from 1000 replicates (grey). (C) Median weekly simulated spillover and human-to-human Nipah cases. (D) Average weekly number of reactive campaigns triggered via spillover detection compared to the estimated seasonal spillover rate (red line).

**
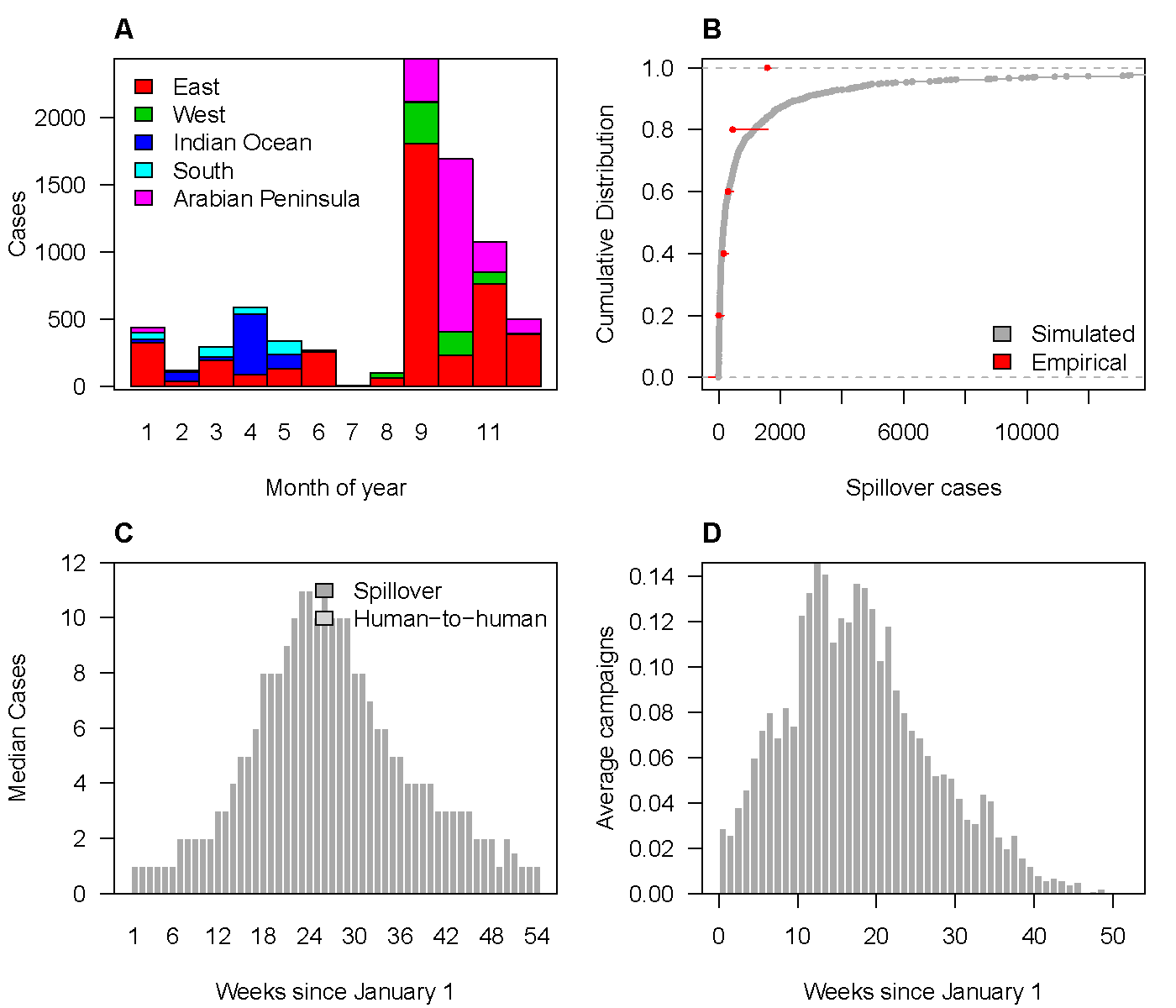
**

**Figure S26. Spillover and reactive vaccination patterns for Rift Valley fever virus (RVFV) within adm1 catchment areas.** (A) Observed monthly RVF spillover cases by region. (B) Annual number of spillovers over the past 5 years (red) and cumulative distribution of simulated annual spillovers from 1000 replicates (grey). (C) Median weekly simulated spillover and human-to-human RVF cases. (D) Average weekly number of reactive campaigns triggered via spillover detection compared to the estimated seasonal spillover rate (red line).

**
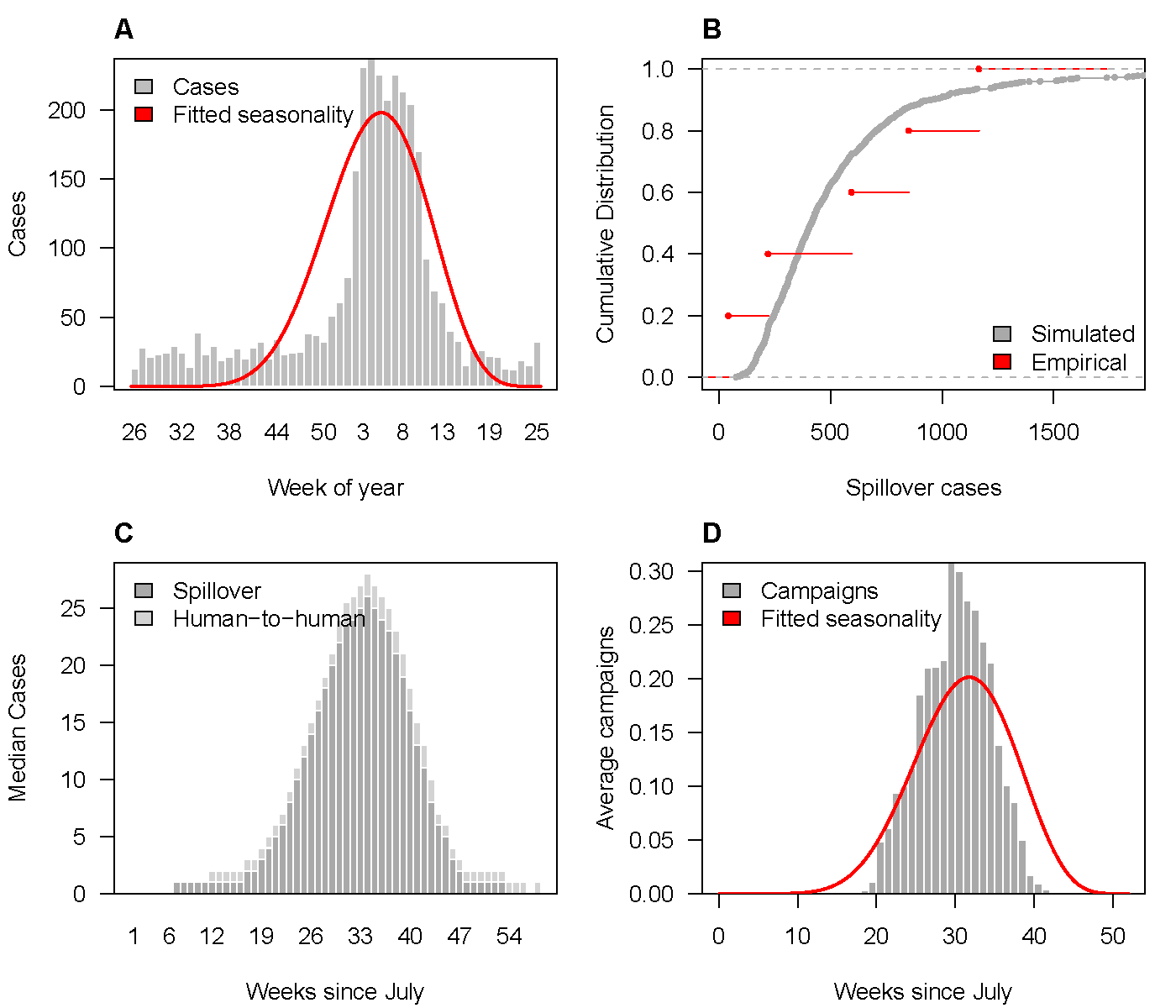
**

**Figure S27. Spillover and reactive vaccination patterns for Lassa fever virus (LASV) within adm1 hospital catchment areas.** (A) Observed weekly Lassa fever spillover cases (grey bars) and estimated seasonal spillover rate (red line). (B) Annual number of spillovers over the past 5 years (red) and cumulative distribution of simulated annual spillovers from 1000 replicates (grey). (C) Median weekly simulated spillover and human-to-human Lassa fever cases. (D) Average weekly number of reactive campaigns triggered via spillover detection compared to the estimated seasonal spillover rate (red line).

**
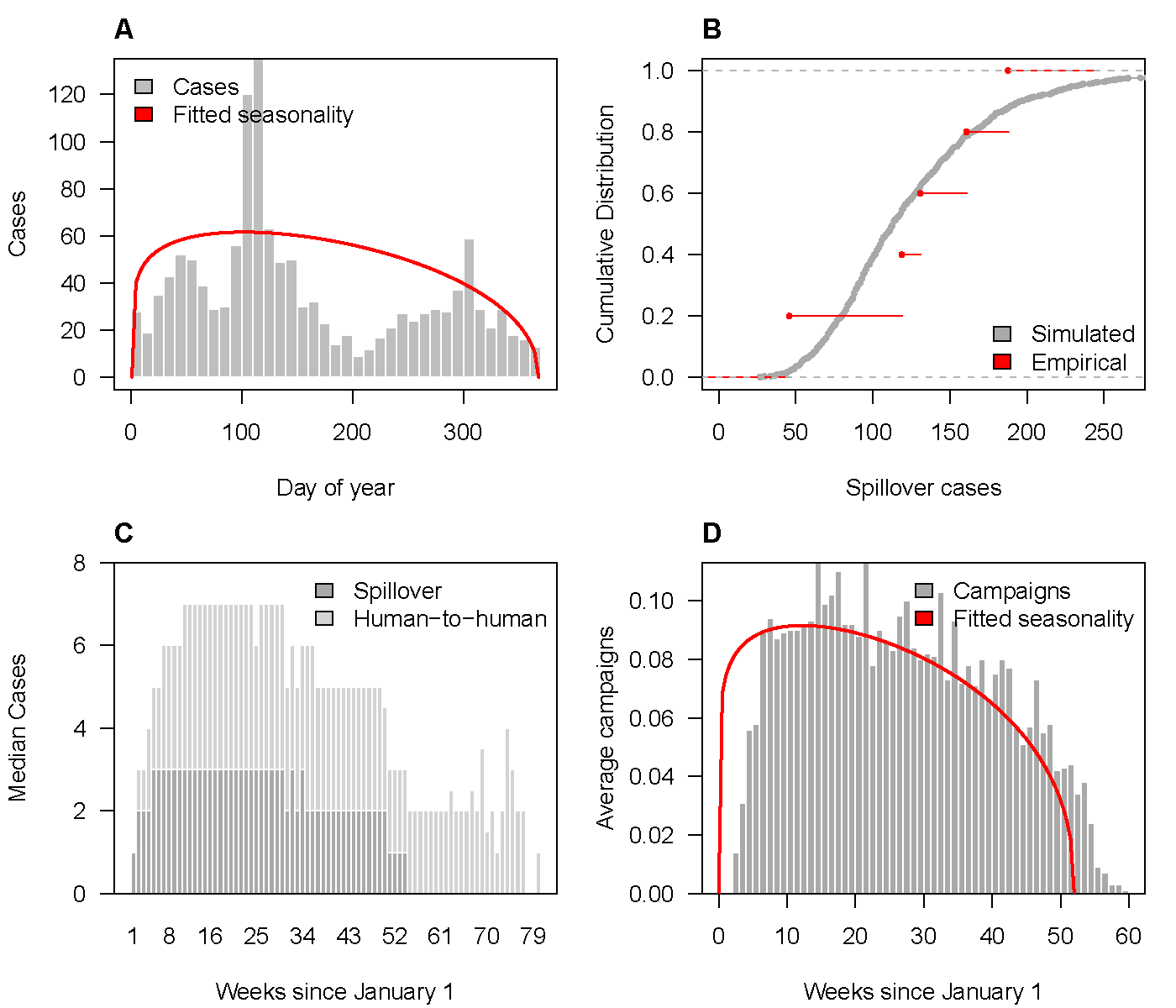
**

**Figure S28. Spillover and reactive vaccination patterns for Middle Eastern respiratory virus (MERS-CoV) within adm1 hospital catchment areas.** (A) Observed weekly MERS spillover cases (grey bars) and estimated seasonal spillover rate (red line). (B) Annual number of spillovers over the past 5 years (red) and cumulative distribution of simulated annual spillovers from 1000 replicates (grey). (C) Median weekly simulated spillover and human-to-human MERS cases. (D) Average weekly number of reactive campaigns triggered via spillover detection compared to the estimated seasonal spillover rate (red line).

**
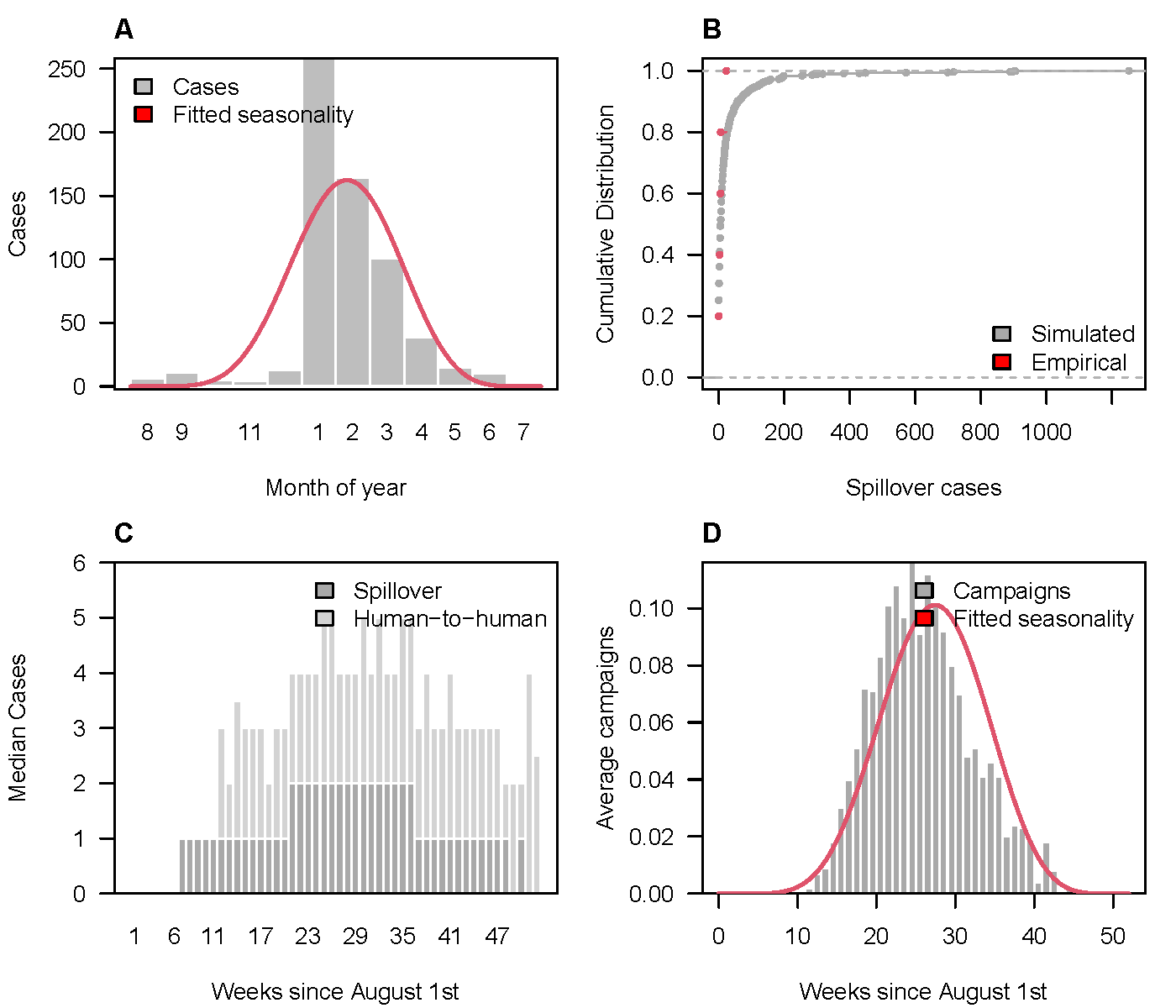
**

**Figure S29. Spillover and reactive vaccination patterns for Nipah virus (NiV) within adm1 hospital catchment areas.** (A) Observed weekly Nipah spillover cases (grey bars) and estimated seasonal spillover rate (red line). (B) Annual number of spillovers over the past 5 years (red) and cumulative distribution of simulated annual spillovers from 1000 replicates (grey). (C) Median weekly simulated spillover and human-to-human Nipah cases. (D) Average weekly number of reactive campaigns triggered via spillover detection compared to the estimated seasonal spillover rate (red line).

**
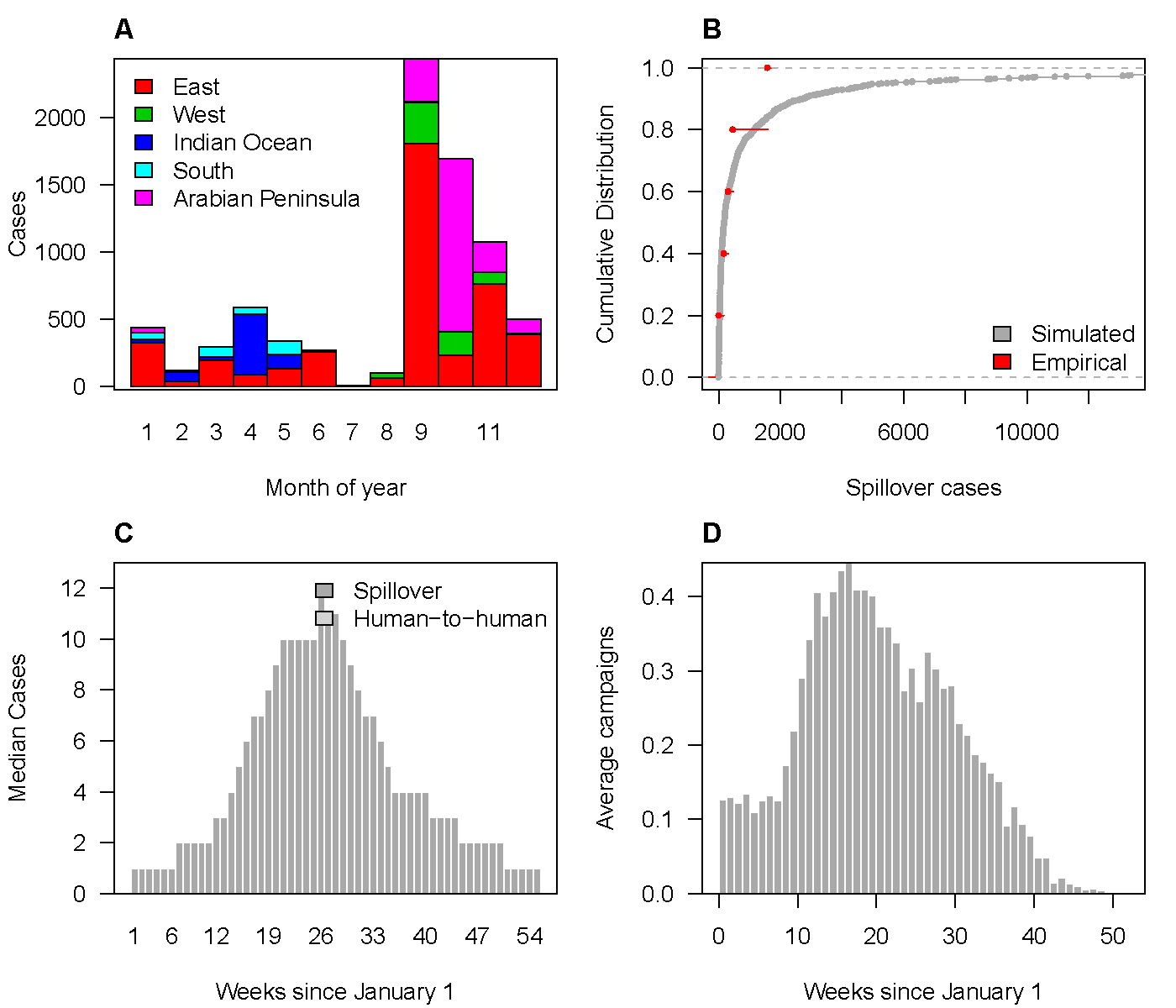
**

**Figure S30. Spillover and reactive vaccination patterns for Rift Valley fever virus (RVFV) within adm1 hospital catchment areas.** (A) Observed monthly RVF spillover cases by region. (B) Annual number of spillovers over the past 5 years (red) and cumulative distribution of simulated annual spillovers from 1000 replicates (grey). (C) Median weekly simulated spillover and human-to-human RVF cases. (D) Average weekly number of reactive campaigns triggered via spillover detection compared to the estimated seasonal spillover rate (red line).

**
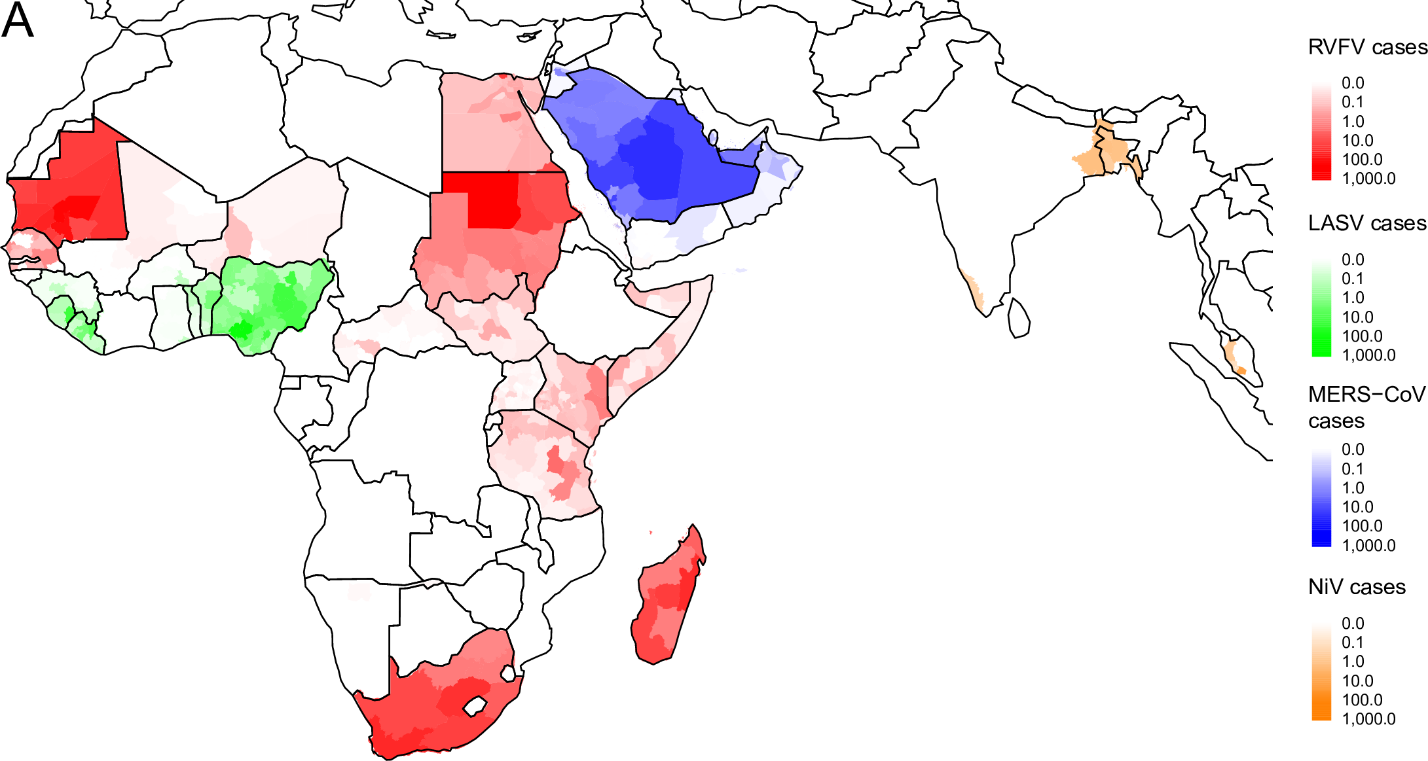
**

**
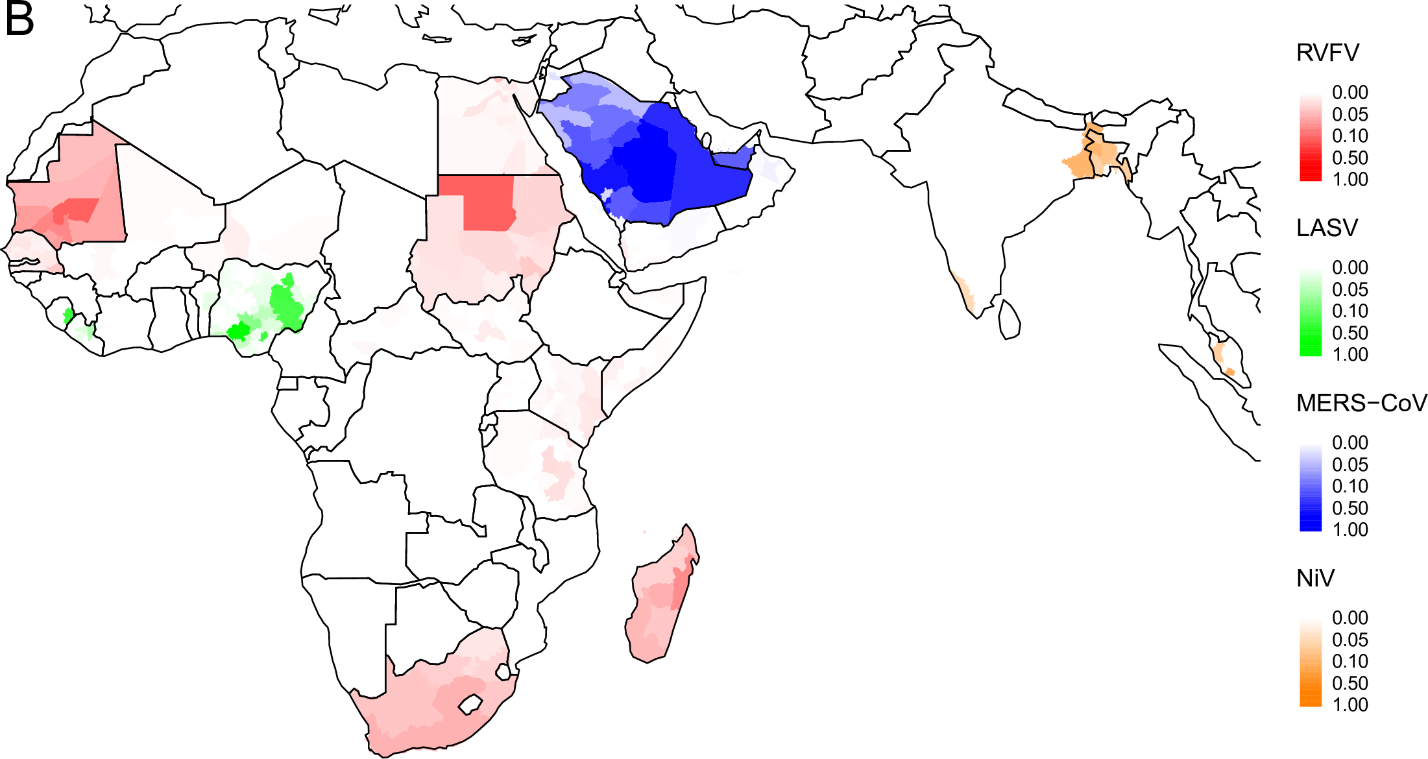
**

**Figure S31. Geographic distribution of spillover cases and reactive vaccination campaigns for adm1 catchment areas.** (A) Geographic distribution of the expected annual number of spillover cases for each pathogen. (B) Probability that a campaign will be triggered.

**
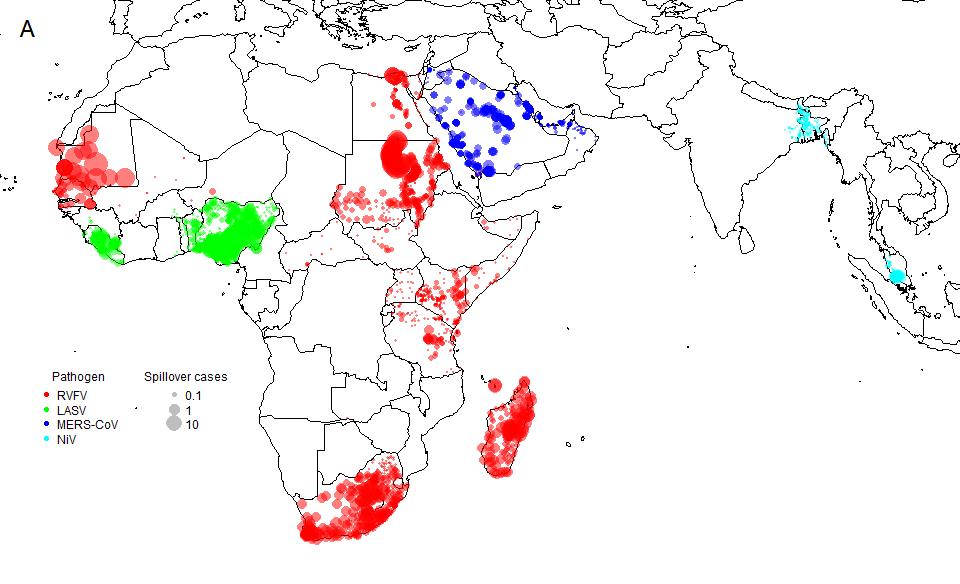
**

**
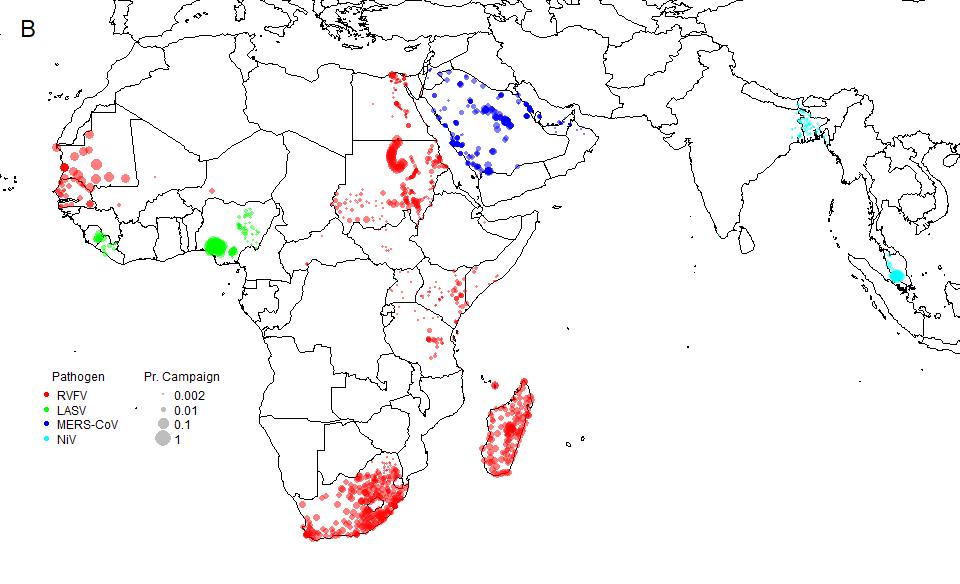
**

**Figure S32. Geographic distribution of spillover cases and reactive vaccination campaigns for adm1 hospital catchment areas.** (A) Geographic distribution of the expected annual number of spillover cases for each pathogen. (B) Proportion of time a campaign will be triggered.

**
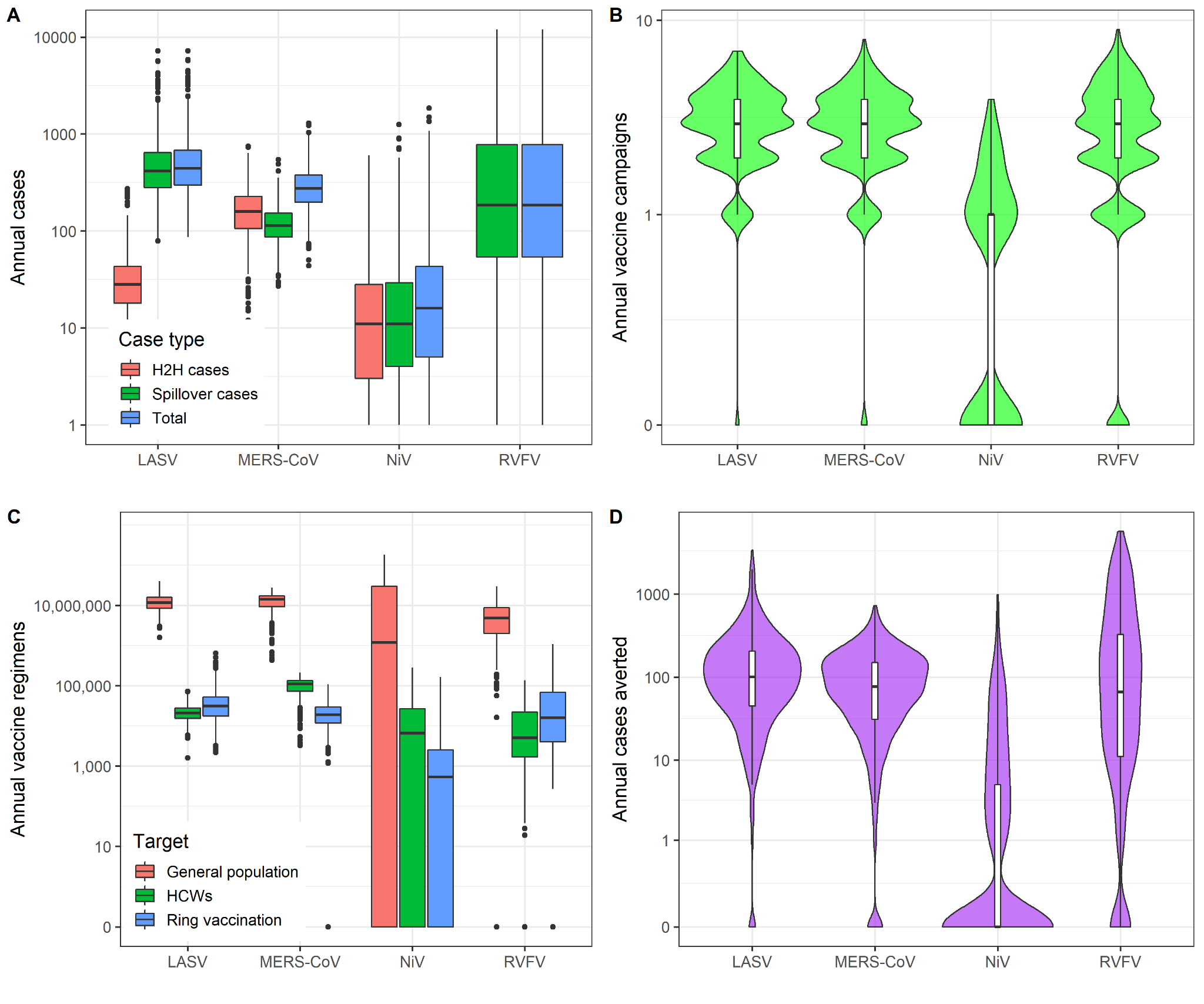
**

**Figure S33. Annual cases and reactive vaccination impacts for adm1 catchment areas.** (A) Annual number of spillover, human-to-human (H2H), and total cases for each pathogen across the entire study region. (B) Annual number of vaccine campaigns that will be triggered due to the outbreak threshold. (C) Number of vaccine regimens required per year for outbreak response when either the general population or healthcare workers (HCWs) only are targeted. (D) Annual number of cases averted via vaccination.

**
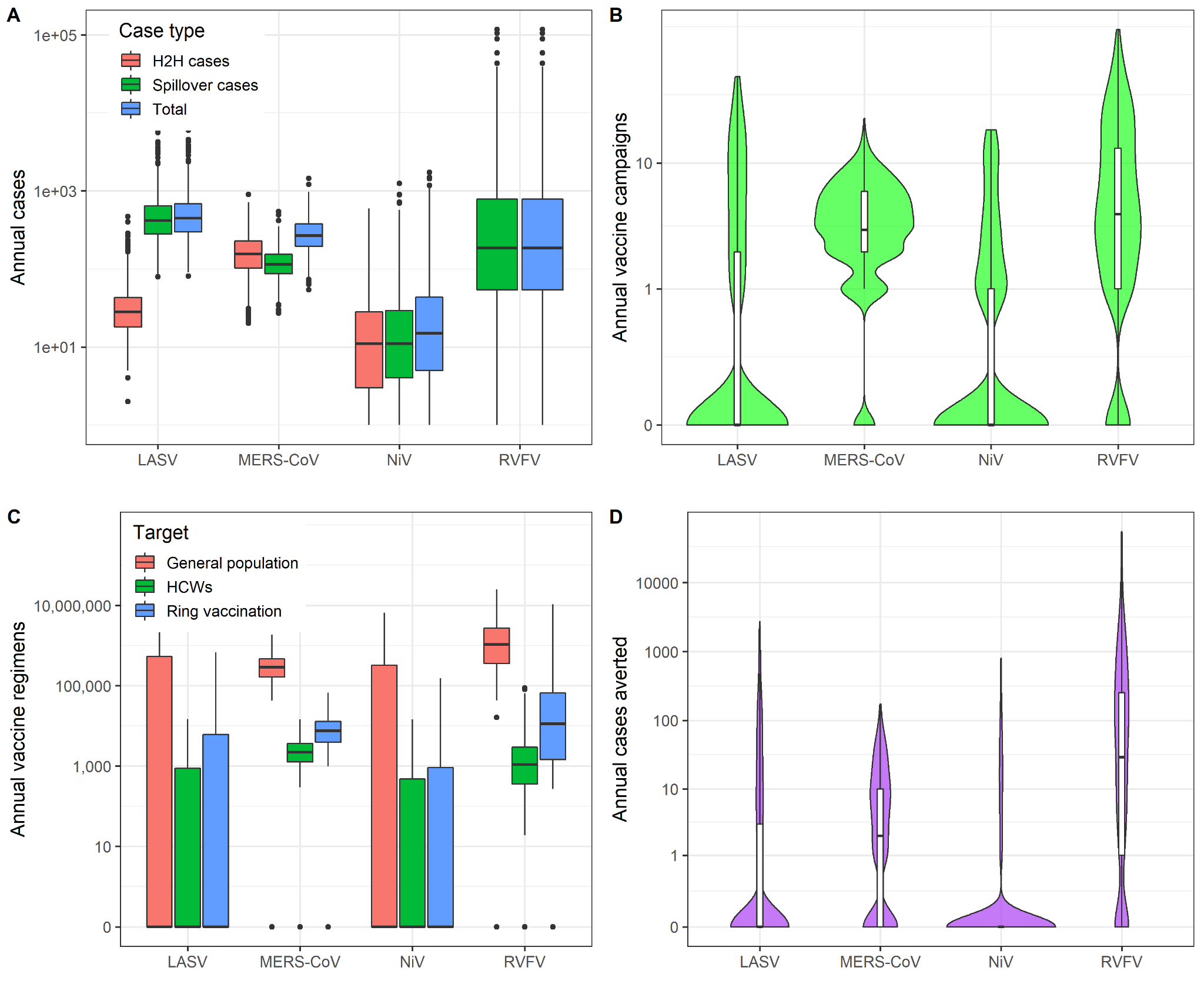
**

**Figure S34. Annual cases and reactive vaccination impacts for adm1 hospital-based catchment areas.** (A) Annual number of spillover, human-to-human (H2H), and total cases for each pathogen across the entire study region. (B) Annual number of vaccine campaigns that will be triggered due to the outbreak threshold. (C) Number of vaccine regimens required per year for outbreak response when either the general population or healthcare workers (HCWs) only are targeted. (D) Annual number of cases averted via vaccination.

**References**

1. Nigeria Centre for Disease Control. Nigeria Centre for Disease Control [Internet]. 2020 [cited 2020 Dec 31]. Available from: https://ncdc.gov.ng/diseases/sitreps/?cat=5&name=An%20update%20of%20Lassa%20fever%20outbreak%20in%20Nigeria

2. Carey DE, Kemp GE, White HA, Pinneo L, Addy RF, Fom AL, et al. Lassa fever. Epidemiological aspects of the 1970 epidemic, Jos, Nigeria. Trans R Soc Trop Med Hyg. 1972;66(3):402–8.

3. Monath TP, Mertens PE, Patton R, Moser CR, Baum JJ, Pinneo L, et al. A hospital epidemic of Lassa fever in Zorzor, Liberia, March-April 1972. Am J Trop Med Hyg. 1973 Nov;22(6):773–9.

4. Bowen GS, Tomori O, Wulff H, Casals J, Noonan A, Downs WG. Lassa fever in Onitsha, East Central State, Nigeria in 1974. Bull World Health Organ. 1975;52(4–6):599–604.

5. E Keane HMG. Lassa fever in Panguma Hospital, Sierra Leone, 1973-6. Br Med J. 1977 May 28;1(6073):1399.

6. ter Meulen J, Lenz O, Koivogui L, Magassouba N, Kaushik SK, Lewis R, et al. Short communication: Lassa fever in Sierra Leone: UN peacekeepers are at risk. Trop Med Int Health. 2001 Jan;6(1):83–4.

7. Ajayi NA. Containing a Lassa fever epidemic in a resource-limited setting: outbreak description and lessons learned from Abakaliki, Nigeria (January–March 2012). Int J Infect Dis. 2013 Nov 1;17(11):e1011–6.

8. Fisher-Hoch SP, Tomori O, Nasidi A, Perez-Oronoz GI, Fakile Y, Hutwagner L, et al. Review of cases of nosocomial Lassa fever in Nigeria: the high price of poor medical practice. BMJ. 1995 Sep 30;311(7009):857–9.

9. Fraser DW, Campbell CC, Monath TP, Goff PA, Gregg MB. Lassa fever in the Eastern Province of Sierra Leone, 1970-1972. I. Epidemiologic studies. Am J Trop Med Hyg. 1974 Nov;23(6):1131–9.

10. Hamblion EL, Raftery P, Wendland A, Dweh E, Williams GS, George RNC, et al. The challenges of detecting and responding to a Lassa fever outbreak in an Ebola-affected setting. Int J Infect Dis. 2018 Jan;66:65–73.

11. ProMED-mail. ProMED-mail [Internet]. 2020 [cited 2020 Dec 31]. Available from: https://promedmail.org/

12. MERS-CoV Cases [Internet]. [cited 2020 Dec 4]. Available from: http://rambaut.github.io/MERS-Tools/cases2.html

13. Ministry of Health - Kingdom of Saudi Arabia [Internet]. 2020 [cited 2020 Dec 31]. Available from: https://www.moh.gov.sa/en/CCC/events/national/Pages/2020.aspx

14. Reeves T, Samy AM, Townsend Peterson A. MERS-CoV geography and ecology in the Middle East: analyses of reported camel exposures and a preliminary risk map. BMC Res Notes. 2015 Dec 18;8(1):1–7.

15. Hsu VP, Hossain MJ, Parashar UD, Ali MM, Ksiazek TG, Kuzmin I, et al. Nipah virus encephalitis reemergence, Bangladesh. Emerg Infect Dis. 2004 Dec;10(12):2082–7.

16. Gurley ES, Montgomery JM, Hossain MJ, Islam MR, Molla MAR, Shamsuzzaman SM, et al. Risk of nosocomial transmission of Nipah virus in a Bangladesh hospital. Infect Control Hosp Epidemiol. 2007 Jun;28(6):740–2.

17. Kumar CPG, Sugunan AP, Yadav P, Kurup KK, Aarathee R, Manickam P, et al. Infections among Contacts of Patients with Nipah Virus, India. Emerg Infect Dis. 2019 May;25(5):1007–10.

18. Nikolay B, Salje H, Hossain MJ, Khan AKMD, Sazzad HMS, Rahman M, et al. Transmission of Nipah Virus - 14 Years of Investigations in Bangladesh. N Engl J Med. 2019 May 9;380(19):1804–14.

19. Tan K-S, Tan CT, Goh KJ. Epidemiological aspects of Nipah virus infection. Neurol J Southeast Asia. 1999;4(1):77–81.

20. Parashar UD, Sunn LM, Ong F, Mounts AW, Arif MT, Ksiazek TG, et al. Case-control study of risk factors for human infection with a new zoonotic paramyxovirus, Nipah virus, during a 1998-1999 outbreak of severe encephalitis in Malaysia. J Infect Dis. 2000 May;181(5):1755–9.

21. Chan KP, Rollin PE, Ksiazek TG, Leo YS, Goh KT, Paton NI, et al. A survey of Nipah virus infection among various risk groups in Singapore. Epidemiol Infect. 2002 Feb;128(1):93–8.

22. Rift Valley Fever in Egypt and other African countries: Historical review, recent outbreaks and possibility of disease occurrence in Egypt. Acta Trop. 2018 May 1;181:40–9.

23. Laughlin LW, Meegan JM, Strausbaugh LJ, Morens DM, Watten RH. Epidemic Rift Valley fever in Egypt: observations of the spectrum of human illness. Trans R Soc Trop Med Hyg. 1979 Jan 1;73(6):630–3.

24. WHO | Rift Valley fever – Gambia. 2018 Feb 26 [cited 2021 May 6]; Available from: http://www.who.int/csr/don/26-february-2018-rift-valley-fever-gambia/en/

25. Centers for Disease Control and Prevention (CDC). Rift Valley Fever -- East Africa, 1997-1998. MMWR Morb Mortal Wkly Rep. 1998 Apr 10;47(13):261–4.

26. Woods CW, Karpati AM, Grein T, McCarthy N, Gaturuku P, Muchiri E, et al. An Outbreak of Rift Valley Fever in Northeastern Kenya, 1997-98. Emerg Infect Dis. 2002 Feb;8(2):138.

27. Centers for Disease Control and Prevention (CDC). Rift Valley Fever Outbreak --- Kenya, November 2006--January 2007. MMWR Morb Mortal Wkly Rep. 2007 Feb 2;56(4):73–6.

28. Jouan A, Le Guenno B, Digoutte JP, Philippe B, Riou O, Adam F. An RVF epidemic in southern Mauritania. Ann Inst Pasteur Virol [Internet]. 1988 [cited 2021 May 6];139(3). Available from: https://pubmed.ncbi.nlm.nih.gov/3207509/

29. Faye O, Diallo M, Diop D, Elmamy Bezeid O, Bâ H, Niang M, et al. Rift Valley Fever Outbreak with East-Central African Virus Lineage in Mauritania, 2003. Emerg Infect Dis. 2007 Jul;13(7):1016.

30. Sow A, Faye O, Ba Y, Ba H, Diallo D, Faye O, et al. Rift Valley Fever Outbreak, Southern Mauritania, 2012. Emerg Infect Dis. 2014 Feb;20(2):296.

31. Bob NS, Bâ H, Fall G, Ishagh E, Diallo MY, Sow A, et al. Detection of the Northeastern African Rift Valley Fever Virus Lineage During the 2015 Outbreak in Mauritania. Open Forum Infect Dis [Internet]. 2017 Jun 16 [cited 2021 May 6];4(2). Available from: https://academic.oup.com/ofid/article-pdf/4/2/ofx087/33605444/ofx087.pdf

32. Sissoko D, Giry C, Gabrie P, Tarantola A, Pettinelli F, Collet L, et al. Rift Valley Fever, Mayotte, 2007–2008. Emerg Infect Dis. 2009 Apr;15(4):568.

33. Métras R, Cavalerie L, Dommergues L, Mérot P, John Edmunds W, Keeling MJ, et al. The Epidemiology of Rift Valley Fever in Mayotte: Insights and Perspectives from 11 Years of Data. PLoS Negl Trop Dis [Internet]. 2016 Jun [cited 2021 May 6];10(6). Available from: https://www.ncbi.nlm.nih.gov/pmc/articles/PMC4917248/

34. Youssouf H, Subiros M, Dennetiere G, Collet L, Dommergues L, Pauvert A, et al. Rift Valley Fever Outbreak, Mayotte, France, 2018–2019. Emerg Infect Dis. 2020 Apr;26(4):769.

35. Centers for Disease Control and Prevention (CDC). Outbreak Summaries: Rift Valley Fever [Internet]. 2020 [cited 2021 May 6]. Available from: https://www.cdc.gov/vhf/rvf/outbreaks/summaries.html

36. FAO. Rift Valley Fever in Niger: Risk Assessment [Internet]. 2017 [cited 2021 May 6]. Available from: http://www.fao.org/3/i7055e/i7055e.pdf

37. Ahmad K. More deaths from Rift Valley fever in Saudi Arabia and Yemen. The Lancet. 2000;356(9239):1422.

38. Sow A, Faye O, Ba Y, Diallo D, Fall G, Faye O, et al. Widespread Rift Valley Fever Emergence in Senegal in 2013–2014. Open Forum Infect Dis [Internet]. 2016 Jul 21 [cited 2021 May 6];3(3). Available from: https://academic.oup.com/ofid/article-pdf/3/3/ofw149/33622067/ofw149.pdf

39. Nderitu L, Lee JS, Omolo J, Omulo S, O’Guinn ML, Hightower A, et al. Sequential Rift Valley Fever Outbreaks in Eastern Africa Caused by Multiple Lineages of the Virus. J Infect Dis. 2010 Dec 14;203(5):655–65.

40. WHO. Weekly Bulletin on Outbreaks and Other Emergencies [Internet]. 2018 [cited 2021 May 6]. Available from: https://apps.who.int/iris/bitstream/handle/10665/273028/OEW27-300606072018.pdf

41. Archer BN, Thomas J, Weyer J, Cengimbo A, Landoh DE, Jacobs C, et al. Epidemiologic Investigations into Outbreaks of Rift Valley Fever in Humans, South Africa, 2008-2011. Emerg Infect Dis [Internet]. 2013 Dec [cited 2021 May 6];19(12). Available from: https://pubmed.ncbi.nlm.nih.gov/29360021/

42. Frame JD, Baldwin JM Jr, Gocke DJ, Troup JM. Lassa fever, a new virus disease of man from West Africa. I. Clinical description and pathological findings. Am J Trop Med Hyg. 1970 Jul;19(4):670–6.

43. Monath TP. Lassa fever and Marburg virus disease. WHO Chron [Internet]. 1974 May [cited 2020 Dec 29];28(5). Available from: https://pubmed.ncbi.nlm.nih.gov/4208764/

44. Mylne AQN, Pigott DM, Longbottom J, Shearer F, Duda KA, Messina JP, et al. Mapping the zoonotic niche of Lassa fever in Africa. Trans R Soc Trop Med Hyg. 2015 Jun 17;109(8):483–92.

45. Khan SH, Goba A, Chu M, Roth C, Healing T, Marx A, et al. New opportunities for field research on the pathogenesis and treatment of Lassa fever. Antiviral Res. 2008 Apr 1;78(1):103–15.

46. Assiri A, Al-Tawfiq JA, Al-Rabeeah AA, Al-Rabiah FA, Al-Hajjar S, Al-Barrak A, et al. Epidemiological, demographic, and clinical characteristics of 47 cases of Middle East respiratory syndrome coronavirus disease from Saudi Arabia: a descriptive study. Lancet Infect Dis. 2013 Sep;13(9):752–61.

47. Cauchemez S, Fraser C, Van Kerkhove MD, Donnelly CA, Riley S, Rambaut A, et al. Middle East respiratory syndrome coronavirus: quantification of the extent of the epidemic, surveillance biases, and transmissibility. Lancet Infect Dis. 2014 Jan;14(1):50–6.

48. Virlogeux V, Park M, Wu JT, Cowling BJ. Association between Severity of MERS-CoV Infection and Incubation Period. Emerg Infect Dis. 2016 Mar;22(3):526–8.

49. Sha J, Li Y, Chen X, Hu Y, Ren Y, Geng X, et al. Fatality risks for nosocomial outbreaks of Middle East respiratory syndrome coronavirus in the Middle East and South Korea. Arch Virol. 2017 Jan;162(1):33–44.

50. Daubney R, Hudson JR, Garnham PC. Enzootic hepatitis or rift valley fever. An undescribed virus disease of sheep cattle and man from east africa. The Journal of Pathology and Bacteriology. 1931;34(4):545–79.

51. Francis T, Magill TP. Rift Valley fever: A report of three cases of laboratory infection and the experimental transmission of the disease to ferrets. J Exp Med. 1935 Aug 31;62(3):433–48.

52. Kitchen SF. Laboratory Infections with the Virus of Rift Valley Fever 1. The American Journal of Tropical Medicine and Hygiene. 1934;s1-14(6):547–64.

53. Findlay GM. Rift valley fever or enzootic hepatitis. Trans R Soc Trop Med Hyg. 1932 Jan 30;25(4):229-IN11.

54. Sabin AB, Blumberg RW. Human infection with Rift Valley fever virus and immunity twelve years after single attack. Proc Soc Exp Biol Med. 1947 Apr;64(4):385–9.

55. Smithburn KC, Mahaffy AF. Rift Valley fever; accidental infections among laboratory workers. J Immunol. 1949 Jun;62(2):213–27.

56. Mundel B, Gear J. Rift valley fever; I. The occurrence of human cases in Johannesburg. S Afr Med J. 1951 Nov 3;25(44):797–800.

57. Hoogstraal H, Meegan JM, Khalil GM, Adham FK. The Rift Valley fever epizootic in Egypt 1977-78. 2. Ecological and entomological studies. Trans R Soc Trop Med Hyg. 1979;73(6):624–9.

58. Ki M. 2015 MERS outbreak in Korea: hospital-to-hospital transmission. Epidemiol Health. 2015 Jul 21;37:e2015033.

59. Park SH, Kim Y-S, Jung Y, Choi SY, Cho N-H, Jeong HW, et al. Outbreaks of Middle East Respiratory Syndrome in Two Hospitals Initiated by a Single Patient in Daejeon, South Korea. Infect Chemother. 2016 Jun;48(2):99–107.

60. Gurley ES, Montgomery JM, Hossain MJ, Bell M, Azad AK, Islam MR, et al. Person-to-person transmission of Nipah virus in a Bangladeshi community. Emerg Infect Dis. 2007 Jul;13(7):1031–7.

61. Bird BH, Ksiazek TG, Nichol ST, Maclachlan NJ. Rift Valley fever virus. J Am Vet Med Assoc. 2009 Apr 1;234(7):883–93.

62. Lo Iacono G, Cunningham AA, Fichet-Calvet E, Garry RF, Grant DS, Khan SH, et al. Using modelling to disentangle the relative contributions of zoonotic and anthroponotic transmission: the case of lassa fever. PLoS Negl Trop Dis [Internet]. 2015 Jan 8 [cited 2020 Dec 29];9(1). Available from: https://pubmed.ncbi.nlm.nih.gov/25569707/

63. Breban R, Riou J, Fontanet A. Interhuman transmissibility of Middle East respiratory syndrome coronavirus: estimation of pandemic risk. Lancet. 2013 Aug 24;382(9893):694–9.

64. Poletto C, Pelat C, Levy-Bruhl D, Yazdanpanah Y, Boelle PY, Colizza V. Assessment of the Middle East respiratory syndrome coronavirus (MERS-CoV) epidemic in the Middle East and risk of international spread using a novel maximum likelihood analysis approach. Euro Surveill [Internet]. 2014 Jun 12;19(23). Available from: http://dx.doi.org/10.2807/1560-7917.es2014.19.23.20824

65. Chowell G, Blumberg S, Simonsen L, Miller MA, Viboud C. Synthesizing data and models for the spread of MERS-CoV, 2013: key role of index cases and hospital transmission. Epidemics. 2014 Dec;9:40–51.

66. Kucharski AJ, Althaus CL. The role of superspreading in Middle East respiratory syndrome coronavirus (MERS-CoV) transmission. Euro Surveill. 2015 Jun 25;20(25):14–8.

67. Cauchemez S, Nouvellet P, Cori A, Jombart T, Garske T, Clapham H, et al. Unraveling the drivers of MERS-CoV transmission. Proc Natl Acad Sci U S A. 2016 Aug 9;113(32):9081–6.

68. Group TWM-CR, The WHO MERS-CoV Research Group. State of Knowledge and Data Gaps of Middle East Respiratory Syndrome Coronavirus (MERS-CoV) in Humans. PLoS Currents [Internet]. 2013; Available from: http://dx.doi.org/10.1371/currents.outbreaks.0bf719e352e7478f8ad85fa30127ddb8

69. Arunkumar G, Chandni R, Mourya DT, Singh SK, Sadanandan R, Sudan P, et al. Outbreak Investigation of Nipah Virus Disease in Kerala, India, 2018. J Infect Dis. 2019 May 24;219(12):1867–78.

70. Lee EC, Wada NI, Grabowski MK, Gurley ES, Lessler J. The engines of SARS-CoV-2 spread. Science. 2020 Oct 23;370(6515):406–7.

71. Tindale LC, Stockdale JE, Coombe M, Garlock ES, Lau WYV, Saraswat M, et al. Evidence for transmission of COVID-19 prior to symptom onset. Elife [Internet]. 2020 Jun 22;9. Available from: http://dx.doi.org/10.7554/eLife.57149

72. McCormick JB, Webb PA, Krebs JW, Johnson KM, Smith ES. A prospective study of the epidemiology and ecology of Lassa fever. J Infect Dis. 1987 Mar;155(3):437–44.

73. Kernéis S, Koivogui L, Magassouba N, Koulemou K, Lewis R, Aplogan A, et al. Prevalence and Risk Factors of Lassa Seropositivity in Inhabitants of the Forest Region of Guinea: A Cross-Sectional Study [Internet]. Vol. 3, PLoS Neglected Tropical Diseases. 2009. p. e548. Available from: http://dx.doi.org/10.1371/journal.pntd.0000548

74. O’Hearn AE, Voorhees MA, Fetterer DP, Wauquier N, Coomber MR, Bangura J, et al. Serosurveillance of viral pathogens circulating in West Africa. Virol J. 2016 Oct 3;13(1):163.

75. Gibb R, Moses LM, Redding DW, Jones KE. Understanding the cryptic nature of Lassa fever in West Africa. Pathog Glob Health. 2017 Sep;111(6):276–88.

76. Müller MA, Meyer B, Corman VM, Al-Masri M, Turkestani A, Ritz D, et al. Presence of Middle East respiratory syndrome coronavirus antibodies in Saudi Arabia: a nationwide, cross-sectional, serological study [Internet]. Vol. 15, The Lancet Infectious Diseases. 2015. p. 559–64. Available from: http://dx.doi.org/10.1016/s1473-3099(15)70090-3

77. Munyua PM, Ngere I, Hunsperger E, Kochi A, Amoth P, Mwasi L, et al. Low-Level Middle East Respiratory Syndrome Coronavirus among Camel Handlers, Kenya, 2019. Emerg Infect Dis. 2021;27(4):1201–5.

78. Bron GM, Strimbu K, Cecilia H, Lerch A, Moore SM, Tran Q, et al. Over 100 years of Rift Valley Fever: a patchwork of data on pathogen spread and spillover. Pathogens. 2021;10(6):708.

79. Chan KP, Rollin PE, Ksiazek TG, Leo YS, Goh KT, Paton NI, et al. A survey of Nipah virus infection among various risk groups in Singapore. Epidemiology & Infection. 2002 Feb;128(1):93–8.

80. Overbosch F, de Boer M, Veldkamp KE, Ellerbroek P, Bleeker-Rovers CP, Goorhuis B, et al. Public health response to two imported, epidemiologically related cases of Lassa fever in the Netherlands (ex Sierra Leone), November 2019. Euro Surveill [Internet]. 2020 Apr;25(15). Available from: http://dx.doi.org/10.2807/1560-7917.ES.2020.25.15.2000265

81. Dudas G, Carvalho LM, Rambaut A, Bedford T. MERS-CoV spillover at the camel-human interface. Elife [Internet]. 2018 Jan 16;7. Available from: http://dx.doi.org/10.7554/eLife.31257

82. Islam MS, Sazzad HMS, Satter SM, Sultana S, Hossain MJ, Hasan M, et al. Nipah Virus Transmission from Bats to Humans Associated with Drinking Traditional Liquor Made from Date Palm Sap, Bangladesh, 2011-2014. Emerg Infect Dis. 2016 Apr;22(4):664–70.
